# Fast computational mutation-response scanning of proteins

**DOI:** 10.7717/peerj.11330

**Published:** 2021-04-21

**Authors:** Julian Echave

**Affiliations:** Instituto de Ciencias Físicas, Escuela de Ciencia y Tecnología, Universidad Nacional de San Martín, San Martín, Buenos Aires, Argentina

**Keywords:** Protein, Mutational response, Compensatory mutations

## Abstract

Studying the effect of perturbations on protein structure is a basic approach in protein research. Important problems, such as predicting pathological mutations and understanding patterns of structural evolution, have been addressed by computational simulations that model mutations using forces and predict the resulting deformations. In single mutation-response scanning simulations, a sensitivity matrix is obtained by averaging deformations over point mutations. In double mutation-response scanning simulations, a compensation matrix is obtained by minimizing deformations over pairs of mutations. These very useful simulation-based methods may be too slow to deal with large proteins, protein complexes, or large protein databases. To address this issue, I derived analytical closed formulas to calculate the sensitivity and compensation matrices directly, without simulations. Here, I present these derivations and show that the resulting analytical methods are much faster than their simulation counterparts.

## Introduction

Protein function is fundamentally related to protein structure. For this reason, insight into protein function can be gained by studying the structural deformations caused by perturbations. This is at the basis of general experimental and theoretical approaches to study proteins. An experimental example is Deep Mutational Scanning, which allows studying the effects of large numbers of mutations (Fowler & Fields, 2014; Livesey & Marsh, 2020). Theoretically, various computational perturbation-response methods have been developed and used to study the effects of ligand binding and mutations (Yilmaz & Atilgan, 2000; Ikeguchi et al., 2005; Zheng & Brooks, 2005; Echave, 2008; Atilgan & Atilgan, 2009).

Ligand binding can be modelled using forces applied to the protein residues involved in binding (Ikeguchi et al., 2005; Atilgan & Atilgan, 2009). This has been used to study various interesting problems. The most straightforward is predicting the conformational change induced by the binding of a ligand, when the binding site is known (Ikeguchi et al., 2005; Atilgan & Atilgan, 2009; Tamura & Hayashi, 2015). A related application is the prediction of ligand-binding sites related to known or desired deformations (Atilgan et al., 2010; Jalalypour et al., 2020). Another important application is the identification of allosteric sites and allosteric communication networks (General et al., 2014; Alfayate et al., 2019; Lake et al., 2020).

Mutations can also be modelled as forces and predicting the resulting responses Echave (2008). Mutation-response computations have been used for various problems. One example is the analysis and prediction of pathological mutations (Nevin Gerek, Kumar & Banu Ozkan, 2013; Tiberti et al., 2018). Another major application is the study of patterns of protein evolutionary divergence (Echave, 2008; Echave & Fernández, 2010; Nevin Gerek, Kumar & Banu Ozkan, 2013; Marcos & Echave, 2020).

In this paper, I focus on mutation-response methods. I consider two cases, mutation-reponse scanning and double mutation-response scanning. In mutation-response scanning, protein sites are scanned over, for each site many random mutations (modelled as forces) are introduced, the resulting deformations are calculated, and deformations are averaged over to obtain a sensitivity matrix, **S** (Echave, 2008; General et al., 2014) (Element *S*_*ij*_ of **S** measures the mean structural deformation of site *i* due to mutations at site *j*.) In double mutation-response scanning, pairs of sites are scanned over, random mutations are introduced, the resulting deformations are calculated, and the minimum deformations are used to calculate a compensation matrix, **D** (Tiberti et al., 2018) (Element *D*_*ij*_ of **D** measures the degree to which mutating site *i* can be compensated by mutating site *j*.) Because they are based on averaging and maximizing over several simulated mutations, I will call the previous methods *simulation-based Mutation-Response Scanning* (sMRS) and *simulation-based Double Mutation-Response Scanning* (sDMRS).

The previous simulation-based methods are not very computationally costly for small to medium proteins. However, the computational cost of sMRS and sDMRS simulations increases with increasing protein size. Therefore, calculations may become prohibitive for very large systems (e.g., supra-molecular complexes, like a ribosome or a virus capsid) or large sets of proteins (e.g., scanning the whole human proteome to detect potential pathological mutations). To alleviate this problem, faster methods are needed.

The purpose of the present paper is to present faster alternatives to sMRS and sDMRS. This article presents two analytical methods, aMRS and aDMRS, that allow, respectively, the calculation of **S** and **D** using closed-formed analytical formulas, without performing simulations. In the following sections, I describe the simulation methods, derive the analytical alternatives, and assess the analytical methods by comparison with their simulation-based counterparts.

## Methods

In the following sections, I derive the formalism of Mutation Response Scanning (MRS) and Double Mutation Response Scanning (DMRS).

### Covariance matrix

At finite temperature the protein fluctuates, sampling an ensemble of conformations. Let a specific backbone conformation be specified by the position vector **r** = (*x*_1_, *y*_1_, *z*_1_, … *x*_*N*_, *y*_*N*_, *z*_*N*_)^*T*^, where (*x*_*i*_, *y*_*i*_, *z*_*i*_) are the Cartesian coordinates of the alpha carbon (*C*_*α*_) of site *i*, *N* is the number of sites, and super-index *T* denotes matrix or vector transposition. The native ensemble can be characterized by the *native structure*, **r**^0^ =〈**r**〉, and by the *covariance matrix*:

(1) $$C\equiv \langle (r-r^{0})(r-r^{0}{)}^{T}\rangle$$

where〈⋯〉is the average over conformations.

The covariance matrix is determined by the protein’s energy landscape. For simplicity, in this work I use the energy function of the Anisotropic Network Model (ANM) (Atilgan et al., 2001). This model represents the protein as a network of amino acids connected by harmonic springs. Specifically, each residue is represented by a single node placed at its *C*_*α*_, and pairs of nodes that are within a cut-off distance *R*_0_ are connected with springs of force-constant *k*. The ANM energy function is

(2) $$V(r)=\frac{1}{2}\sum_{ij}k(∥r_{j}-r_{i}∥-∥r_{j}^{0}-r_{i}^{0}∥{)}^{2}$$

where **r**_*x*_ is the position vector of node *x*, **r**^0^_*x*_ its equilibrium position, *k* is the spring force constant, and the sum runs over all contacts *ij*.

The covariance matrix can be derived from Eq. (2). First, a second-order Taylor expansion of (2) leads to

(3) $$V(r)\approx \frac{1}{2}(r-r^{0}{)}^{T}K(r-r^{0})$$

where $K={\left(d^{2}Vdr^{2}\right)}_{r=r^{0}}$ is the Hessian matrix. Then, assuming a Boltzmann distribution of conformations *ρ*(**r**) = *e*^−*V*(**r**)/*k*_*B*_^^*T*^ with *V*(**r**) given by (3), it follows that

(4) $$C=k_{B}TK^{-1}$$

where *k*_*B*_ is Boltzmann’s constant, *T* the absolute temperature, and **K**^−1^ is the Hessian’s pseudo-inverse (**K** is not invertible because it has 6 zero eigenvalues corresponding to rotations and translations). Given a protein of known native structure **r**^0^, and parameters *R*_0_ and *k*, **K** is calculated differentiating (2), then **C** is obtained using (4).

### Linear response approximation

The covariance matrix determines the conformational shift that results from applying a force to one or more protein atoms. An arbitrary force can be represented by a vector **f** with one component for each of the coordinates that represent the protein’s conformation. For small **f**, the structural response can be calculated using the Linear Response Approximation (LRA) (Ikeguchi et al., 2005; Echave, 2008):

(5) $$\Delta r^{0}=\frac{C}{k_{B}T}f$$

Equation (5) allows the prediction of the effect of any given force **f** with the sole knowledge of **C**.

### Mutation-response scanning

The aim of Mutation-Response Scanning (MRS) is to analyse how protein structure responds to point mutations. In the methods that I consider here, given a protein, mutations are modelled using forces, the resulting structural responses are calculated using the Linear Response Approximation, and these responses are averaged over mutations to calculate a sensitivity matrix **S** that quantifies the mutation-response patterns.

#### Mutations as forces

Point mutations can be modelled by forcing the contacts of the mutated site (Echave, 2008). Let *j* be the site to mutate, *C*(*j*) be the set of contacts of *j*, and *jl* the contact between *j* and *l*. Then, a mutation is modelled by applying a force

(6) $$f(j)=\sum_{jl\in C(j)}f(jl)$$

where **f**(*jl*) is the force applied to contact *jl*. Let *f*(*jl*) be a scalar and **e**_*jl*_ a unit vector directed from *j* to *l*. Then, **f**(*jl*) consists of a force *f*(*jl*)**e**_*jl*_ applied to *l*, plus a reaction force −*f*(*jl*)**e**_*jl*_ applied to *j*, and no force applied to other sites.

A random mutation at site *j* is modelled by picking independent random numbers *f*(*jl*) and building **f**(*jl*) and **f**(*j*) (Eq. (6)). Following previous work (Echave, 2008; Echave & Fernández, 2010; Marcos & Echave, 2020), I use

(7) $$f(jl)∼N(0,\sigma^{2})$$

Thus, the contact forces are picked from independent identical normal distributions.

#### Sensitivity matrix, S

What is the effect on a site *i* of mutating a site *j*? Consider a random mutation at site *j*, represented by a force **f**(*j*). Then, from (5), the structural deformation due to this mutation is given by

(8) $$\Delta r^{0}(j)=\frac{C}{k_{B}T}f(j)$$

Δ**r**^0^(*j*) can be written:

(9) $$\Delta r^{0}(j)=\left(\begin{array}{c} \Delta r_{1}^{0}(j)\\ \vdots \\ \Delta r_{N}^{0}(j) \end{array}\right)$$

where Δ**r**^0^_*i*_(*j*) is the 3 × 1 column vector that contains the change in Cartesian coordinates of site *i* caused by mutation **f**(*j*) applied to site j. Therefore, the magnitude of the effect of the mutation on the structure of site *i* may be quantified by the Euclidean norm ||Δ**r**^0^_*i*_(*j*)^2^||.

The *sensitivity matrix* **S** is the matrix with elements

(10) $$S_{ij}=\left\langle {||\Delta r_{i}^{0}(j)||}^{2}\right\rangle$$

where *i* is the response site, *j* the mutated site, and〈⋯〉stands for averaging over mutations. *S*_*ij*_ represents the structural response of site *i* averaged over mutations at site *j*. Mutation-response scanning is the calculation of the sensitivity matrix **S** defined by 10.

#### Simulation-based mutation-response scanning

The sensitivity matrix **S** can be obtained using the simulation-based Mutation-Response Scanning method, sMRS. Given a protein’s pdb file, this numerical method proceeds as follows.

1. **Set parameters.** Set parameters *k* and *R*_0_ of the ANM model, parameter *σ* used to generate forces (Eq. (7)), and a desired number of mutations to apply to each site, *M*.
2. **Calculate the covariance matrix.** Read protein coordinates from the pdb file, for all pairs of sites calculate *C*_*α*_ − *C*_*α*_ distances, compare them with *R*_0_ to define contacts, then calculate the elastic network’s matrix **K** using (2) and (3). Finally, invert this matrix to calculate **C** using (4).
3. **Generate mutational forces.** For each site *j*, generate *μ* = 1 ⋯ *M* mutational force vectors **f**(*j*, *μ*) using (6) and (7).
4. **Calculate mutational deformations.** For each mutational force **f**(*j*,*μ*), calculate the resulting response Δ**r**^0^_*i*_(*j*, *μ*).
5. **Calculate the sensitivity matrix.** Average the deformations Δ**r**^0^_*i*_(*j*, *μ*) over mutations *μ* to obtain element *S*_*ij*_ of the sensitivity matrix **S**, according to (10).

#### Analytical formula for the sensitivity matrix

In this section, I derive an analytical formula that allows the direct calculation of the sensitivity matrix, **S**, without performing simulations.

The first step is to consider the deformation caused by forcing a single contact. Let **f**(*jl*) be a force applied along contact *jl*, composed by a force *f*(*jl*)**e**_*jl*_ applied to *l* and a reaction force −*f*(*jl*)**e**_*jl*_ applied to *j*. Replacing **f**(*jl*) into (5) and using (9), leads to

(11) $$\Delta r_{i}^{0}(jl)=\left(C_{il}-C_{ij}\right)e_{jl}f(jl)$$

where Δ**r**^0^_*i*_(*jl*) is the structural shift of site *i* caused by **f**(*jl*) and **C**_*xy*_ is the 3 × 3 block of **C** corresponding to the covariance between sites *x* and *y*.

Second, the deformation resulting from mutating a site is the sum of the deformations caused by forcing its contacts. From (6), (8), and (9), it follows that

(12) $$\Delta r_{i}^{0}(j)=\sum_{jl\in C(j)}\Delta r_{i}^{0}(jl)$$

where Δ**r**^0^_*i*_(*j*) is the shift of *i* due to mutating *j* and the sum runs over all contacts of *j*. Replacing (11) into (12), leads to

(13) $$\Delta r_{i}^{0}(j)=\sum_{jl\in C(j)}\left(C_{il}-C_{ij}\right)e_{jl}f(jl)$$

Finally, an analytical formula for the direct calculation of the sensitivity matrix may be derived. Replacing (13) into (10), leads to

(14) $$\begin{array}{cc} S_{ij}\equiv & \left\langle ∥\Delta r_{i}^{0}(j){∥}^{2}\right\rangle \\ = & \sum_{jl\in C(j)}\sum_{jk\in C(j)}\Delta r_{i}{\left(jk\right)}^{T}\Delta r_{i}(jl)\\ = & \sum_{jk\in C(j)}\sum_{jl\in C(j)}\langle f(jk)f(jl)\rangle e_{jk}^{T}{\left(C_{ik}-C_{ij}\right)}^{T}(C_{il}-C_{ij})e_{jl} \end{array}$$

where〈⋯〉stands for averaging over mutations at *j*. Since *f*(*jl*) ∼ *N*(0, *σ*^2^) are independent identically distributed random variables (“Mutations as forces”), it follows that

(15) $$\langle f(jk)f(jl)\rangle =\sigma^{2}\delta_{jk,jl}$$

where *δ*_*xy*_ is the Kronecker delta, which is 1 for *x* = *y* and 0 otherwise. Therefore, replacing (15) into (14), leads to

(16) $$S_{ij}=\sigma^{2}\sum_{jl\in C(j)}e_{jl}^{T}(C_{il}-C_{ij}{)}^{T}(C_{il}-C_{ij})e_{jl}$$

This equation allows the calculation of the sensitivity matrix.

#### Analytical mutation-response scanning

The analytical Mutation-Response Scanning method, aMRS calculates the sensitivity matrix **S** using the analytical formula (16). Given a protein’s pdb file, this method proceeds as follows.

1. **Set parameters.** Set the parameters *k* and *R*_0_ of the ANM model, and the parameter *σ* that defines the distribution of forces (Eq. (7)).
2. **Calculate the covariance matrix.** Read protein coordinates from the pdb file, for all pairs of sites calculate *C*_*α*_ − *C*_*α*_ distances, compare them with *R*_0_ to define contacts, then calculate the elastic network’s matrix **K** using (2) and (3). Finally, invert this matrix to calculate **C** using (4).
3. **Calculate the sensitivity matrix.** Calculate the elements *S*_*ij*_ of the sensitivity matrix **S** using (16).

### Double mutation-response scanning

The aim of Double Mutation-Response Scanning (DMRS) is to analyse how protein structure responds to pairs of point mutations. Just as for the MRS methods described above, the DMRS methods that I consider in this section model mutations using forces and calculate structural responses using the Linear Response Approximation. These responses are used to calculate a compensation matrix **D** that quantifies the degree of structural compensation between pairs of mutations.

#### Compensation matrix

In this subsection, I define the compensation matrix that DMRS aims to calculate. Let Δ**r**^0^(*iμ*) be the structural response to a mutation *μ* at site *i*, and Δ**r**^0^(*jν*) be the structural response to a mutation *ν* at *j*. The deformation due to introducing both mutations is given by

(17) $$\Delta r^{0}(i\mu ,j\nu )=\Delta r^{0}(i\mu )+\Delta r^{0}(j\nu )$$

and the magnitude of this deformation is given by

(18) $$∥\Delta r^{0}(i\mu ,j\nu ){∥}^{2}=∥\Delta r^{0}(i\mu ){∥}^{2}+∥\Delta r^{0}(j\nu ){∥}^{2}+2\Delta r^{0}(i\mu {)}^{T}\Delta r^{0}(j\nu )$$

The first two terms are positive, but the third term may be positive or negative. When the third term is negative, the mutations will compensate each other. Given a first mutation *iμ*, the maximum compensation due to a second mutation at *j* is obtained when Δ**r**^0^(*iμ*)^*T*^*Δ***r**^0^(*jν*) is minimum. Therefore, the degree of compensation may be quantified by $\underset{\nu }{\min}\Delta r^{0}(i\mu {)}^{T}\Delta r^{0}(j\nu )$. For mutations modelled as forces, this is equal to minus the maximum, because if a force maximizes the dot-product, the opposite force, which is as likely, minimizes it. Therefore, to keeps things positive, it is convenient to define the compensating power of *j* by $\underset{\nu }{\max}[\Delta r^{0}(i\mu {)}^{T}\Delta r^{0}(j\nu ){]}^{2}$. With the help of this equation, I define a compensation matrix, **D**, with elements *D*_*ij*_ given by

(19) $$D_{ij}=\left\langle {max}_{\nu }{\left[\Delta r^{0}(i\mu {)}^{T}\Delta r^{0}(j\nu )\right]}^{2}\right\rangle \mu^{\frac{1}{2}}$$

where〈⋯〉_*μ*_ is the average over *μ*. *D*_*ij*_ is a positive number that quantifies the degree to which mutating *j* can compensate the structural effect of mutating *i*.

#### Forces for double mutation-response scanning

The choice of forces used to model mutations in “Mutations as forces” is not appropriate for calculating the compensation matrix because the maximum involved is ill defined. The value of Δ**r**^0^(*iμ*)^*T*^Δ**r**^0^(*jν*) is proportional to the lengths of force vectors **f**(*iμ*) and **f**(*jμ*). Defined as described in “Mutations as forces”, the lengths of these vectors may become arbitrarily large, making the maximum in (19) infinite. To fix this, I apply the additional constraint

(20) $$∥f(x){∥}^{2}=\sigma^{2}CN(x)$$

where *σ*^2^ is the parameter used to define contact forces (see Eq. (7)) and *CN*(*x*) is the number of contacts of site *x*. In practice, this is achieved by picking the forces as before, then renormalizing them. The norm of these forces is finite and the maximum of (19) is well defined.

#### Simulation-based double mutation-response scanning

The compensation matrix may be obtained using the method simulation-based Double Mutation-Response Scanning, sDMRS, which proceeds as follows.

1. **Set parameters.** Set parameters *k* and *R*_0_ of the ANM model, parameter *σ* used to generate forces (Eq. (7)), and a desired number of mutations to apply to each site, *M*.
2. **Calculate the covariance matrix.** Read protein coordinates from the pdb file, for all pairs of sites calculate *C*_*α*_ − *C*_*α*_ distances, compare them with *R*_0_ to define contacts, then calculate the elastic network’s matrix **K** using (2) and (3). Finally, invert this matrix to calculate **C** using (4).
3. **Generate mutational forces.** For each site *i*, generate *μ* = 1 ⋯ *M* mutational force vectors **f**(*iμ*) using (6), (7), and (20).
4. **Calculate mutational deformations.** For each mutational force **f**(*iμ*), calculate the resulting response Δ**r**^0^(*iμ*).
5. **Calculate the compensation matrix.** For each pair (*iμ*,*jν*), calculate Δ**r**^0^(*iμ*)^*T*^Δ**r**^0^(*jν*), maximize over *ν*, and average over *μ* to obtain the elements of the compensation matrix **D**, according to (19).

#### Analytical formula for the compensation matrix

In this section, I derive an analytical formula that allows the direct calculation of the compensation matrix, **D**, without performing simulations.

The first step is to consider the overlap between two deformations, Δ**r**^0^(*i*)^*T*^Δ**r**^0^(*j*). Consider two mutations, at sites *i* and *j*, represented by forces **f**(*i*) and **f**(*j*), respectively. From (6) and (8), it follows that

(21) $$\begin{array}{cc} \Delta r^{0}(i)= & \sum_{ik\in C(i)}(C_{k}-C_{i})e_{ik}f(ik)\\ \Delta r^{0}(j)= & \sum_{jl\in C(j)}(C_{l}-C_{j})e_{jl}f(jl) \end{array}$$

where Δ**r**^0^(*x*) is the protein’s deformation due to mutating site *x*, **C**_*x*_ is the 3 *N* × 3 block of **C** with the 3 columns corresponding to site *x*, and *f*(*xy*) is the scalar force applied to contact *xy*. From (21), the overlap between two deformations is given by

(22) $$\Delta r^{0}(i{)}^{T}\Delta r^{0}(j)=\sum_{ik\in C(i)}\sum_{jl\in C(j)}f(ik)f(jl)e_{ik}^{T}(C_{k}-C_{i}{)}^{T}(C_{l}-C_{j})e_{jl}$$

For simplicity of notation, it is convenient to rewrite this equation in matrix form:

(23) $$\Delta r^{0}(i{)}^{T}\Delta r^{0}(j)=f(i{)}^{T}A_{ij}f(j)$$

where **f**(*i*) is a column vector whose elements are the *CN*(*i*) contact forces *f*(*ik*), **f**(*j*) is the column vector with *CN*(*j*) elements *f*(*jl*), and **A**_*ij*_ is a matrix of size *CN*(*i*) × *CN*(*j*) with elements

(24) $$A_{ik,jl}\equiv e_{ik}^{T}(C_{k}-C_{i}{)}^{T}(C_{l}-C_{j})e_{jl}$$

At this point it is easy to derive a formula for the compensation matrix. The maximum of ${\left[\Delta r^{0}{\left(i\right)}^{T}\Delta r^{0}(j)\right]}^{2}$, subject to the constraint **f**(*j*)^2^ = *σ*^2^ *CN*(*j*) (Eq. (20)) can be shown to be

(25) $$\max{\left[\Delta r^{0}{\left(i\right)}^{T}\Delta r^{0}(j)\right]}^{2}=CN(j)f(i{)}^{T}A_{ij}A_{ij}^{T}f(i)$$

Then, replacing (25) into (19), and using (15), leads to:

(26) $$D_{ij}=\sigma^{2}\sqrt{CN(j)TrA_{ij}A_{ij}^{T}}$$

where Tr is the trace operator. This equation allows the calculation of the compensation matrix.

#### Analytical double mutation-response scanning

The analytical Double Mutation-Response Scanning method, aDMRS, calculates the compensation matrix **D** using the analytical formula (26). Given a protein’s pdb file, this method proceeds as follows.

1. **Set parameters.** Set the parameters *k* and *R*_0_ of the ANM model, and the parameter *σ* that defines the distribution of forces (Eq. (7)).
2. **Calculate the covariance matrix.** Read protein coordinates from the pdb file, for all pairs of sites calculate *C*_*α*_ − *C*_*α*_ distances, compare them with *R*_0_ to define contacts, then calculate the elastic network’s matrix **K** using (2) and (3). Finally, invert this matrix to calculate **C** using (4).
3. **Calculate the compensation matrix.** Calculate the elements *D*_*ij*_ of the compensation matrix **D** using (26).

### Implementation

In the present work, sMRS (Simulation-based Mutation-Response Scanning), aMRS (Analytical Mutation-Response Scanning), sDMRS (Simulation-based Double Mutation-Response Scanning), and aDMRS (Analytical Double Mutation-Response Scanning) were implemented using the R language. As much as possible, the code was optimised by using the linear algebra functions of the BLAS and LAPACK packages. For implementation details see available code.

### Parameters

The parameter values used in the present paper are *R*_0_ = 12.5 *Å*, *k* = 1/*Å* ^2^, and *σ* = 0.3/*Å*. With the chosen *R*_0_ value, previous work found good agreement between predicted and empirical structural deformations Marcos & Echave (2020). Regarding *k*, energy units are arbitrarily chosen so that *k* = 1/*Å*^2^. The precise values of *k* and *σ* do not affect the present results because they have a mere scaling effect on the sensitivity matrix and the compensation matrix (It can easily be proved that both matrices are proportional to $\frac{\sigma^{2}}{k^{2}}$).

### Dataset

Table 1 summarises the dataset used to assess the methods developed in this work. The structure files for the calculations were obtained from the Protein Data Bank for d2l8ma and d2acya, and from the Homstrad database for the other proteins (Stebbings & Mizuguchi, 2004). I use the 8 Homstrad proteins because mutation-response simulations were tested against empirical data for these proteins in a recent study (Marcos & Echave, 2020). I added the other two proteins, with which I am familiar from other studies, to complete the dataset: d2acya to have a second representative of the alpha & beta SCOP structural class and 2l8ma to add a large protein to the dataset.

**Table 1:** Protein data set.

domain	family	class	*N*
d1lcka1	SH3 domain	All beta	54
d1ntxa	Snake venom toxins	Small	60
d1fxla2	Canonical RNA-binding domain	Alfa & beta	82
d1bxva	Plastocianine/Azurin-like	All beta	91
d2acya	Acyl-phosphatase-like	Alpha & beta	98
d1jiaa	Vertebrate Phospholipase A2	All alpha	122
d1hmta	Fatty acid binding protein-like	All beta	131
d1a4fb	Globines	All alpha	146
d1mcta	Eukaryotic proteases	All beta	223
d2l8ma	Cytochrome P450	All alpha	405

**Note:**

Columns show, in order, protein domain id, family, and structural class according to the SCOP classification (Murzin et al., 1995), and protein length *N*.

## Results

### Mutation-response scanning

This section assesses the analytic Mutation-Response Scanning method (aMRS) by comparison with the simulation-based Mutation-Response Scanning method (sMRS). These methods were described in detail in “Methods”. Briefly, for a given protein, an sMRS simulation consists in subjecting each of the protein sites *j* to *M* mutations, calculating the resulting structural deformation of each site *i*, and averaging these deformations over mutations to obtain the elements *S*_*ij*_ of a sensitivity matrix **S** (see “Simulation-based Mutation-Response Scanning”). The analytical method, aMRS, calculates **S** using the closed analytical expression Eq. (16), avoiding the need of simulations (see “Analytical Mutation-Response Scanning”). Methods are compared on the proteins of Table 1.

#### sMRS converges rapidly towards aMRS

I compare aMRS with sMRS for the proteins of Table 1. The point of this work is to assess whether the analytical method is faster than the simulation method. However, since the calculations performed with the simulation method depend on the number of mutations per site, *M*, before addressing computational cost, I consider the convergence of sMRS calculations.

**Figure 1:**
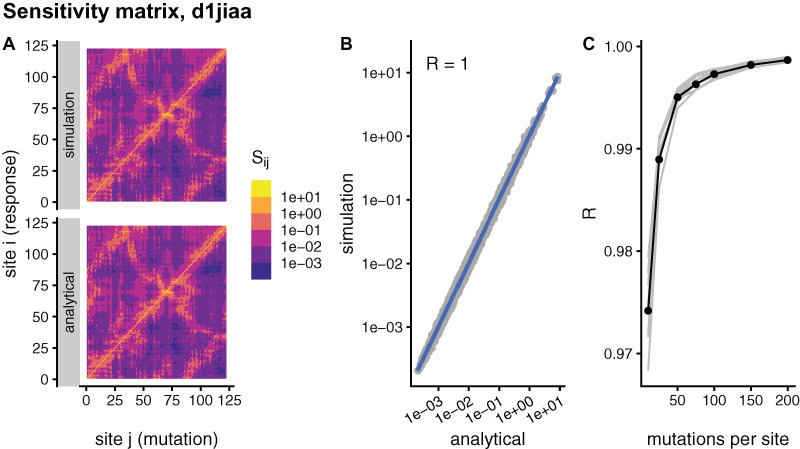
Comparison between sMRS and aMRS sensitivity matrices. Results shown for Phospholipase A2 (d1jiaa). The sensitivity matrix S has elements *S_ij_* that measure the structural shift of site *i* averaged over mutations at site *j*. sMRS is a simulation-based Mutation Response Scanning method that calculates S by averaging over simulated point mutations. aMRS is an analytical method that calculates S using a closed formula. (A) sMRS response matrix obtained by averaging over 200 mutations (simulation) compared with the aMRS matrix (analytical). (B) Scatterplot of the sMRS vs. aMRS matrix elements of A. (C) Convergence of sMRS with increasing number of mutations per site. In C the d1jiaa case is shown with black lines and points, and the other 9 proteins studied are shown with grey lines. Matrix elements Si j are normalised so that their average is 1. Logarithmic scale is used in A and B and *R* is the Pearson correlation coefficient between the log-transformed sMRS and aMRS matrices.

Theoretically, sMRS and aMRS are equivalent ways of calculating the sensitivity matrix **S**. Specifically, in the limit of an infinite number of mutations per site, M→∞, the sMRS **S** should converge towards the aMRS **S**. To study this convergence, Fig. 1, compares simulated and analytical matrices for the example case of Phospholipase A2 (SCOP id d1jiaa) (Similar figures for the other proteins studied can be found in Supplemental_info.pdf). For the d1jiaa example, sMRS converges rapidly towards the aMRS matrix as *M* increases (Fig. 1C), so that the sMRS matrix calculated with *M* = 200 is very similar to the aMRS matrix (Fig. 1A and Fig. 1B).

For the other proteins the results are similar. Thus, for all cases the sMRS matrix converges rapidly towards the aMRS matrix (see grey lines of Fig. 1C). For *M* = 200, the correlation coefficient between sMRS and aMRS matrices is 1.00 for all proteins (Table 2). Thus, the sMRS sensitivity matrix converges rapidly with increasing *M*, so that with *M* = *O*(10^2^) it is very similar to the aMRS matrix.

**Table 2:** aMRS vs. sMRS summary.

protein	*N*	*t*_sMRS_	*t*_aMRS_	R	*R*_*i*_	*R*_*j*_
d1lcka1	54	6.26	0.03	1.00	1.00	0.99
d1ntxa	60	7.18	0.05	1.00	1.00	1.00
d1fxla2	82	11.22	0.07	1.00	1.00	1.00
d1bxva	91	12.44	0.07	1.00	1.00	0.97
d2acya	98	18.08	0.07	1.00	1.00	0.98
d1jiaa	122	18.77	0.12	1.00	1.00	0.99
d1hmta	131	21.16	0.11	1.00	1.00	0.99
d1a4fb	146	26.02	0.18	1.00	1.00	0.99
d1mcta	223	54.08	0.37	1.00	1.00	0.99
d2l8ma	405	180.91	1.47	1.00	1.00	0.99

**Note:**

N: protein length; *t*_sMRS_: CPU time of sMRS in seconds; *t*_aMRS_: CPU time of aMRS in seconds. Convergence measures at *M* = 200 mutations per site: *R*: correlation coefficient between sMRS and aMRS sensitivity matrices; *R*_*i*_: correlation between sensitivity profiles; *R*_*j*_: correlation between influence profiles.

To further assess convergence, I consider sMRS and aMRS profiles. Site-dependent profiles are obtained by averaging the sensitivity matrix over rows or columns. Averaging over rows leads to an *influence profile*, with elements $S_{j}\equiv 1N\sum_{i}^{N}S_{ij}$ that measure the average influence of mutating *j*. Averaging over columns leads to a *sensitivity profile*, with elements $S_{i}\equiv 1N\sum_{j}^{N}S_{ij}$ that measure the sensitivity of site *i* with respect to mutations elsewhere.

**Figure 2:**
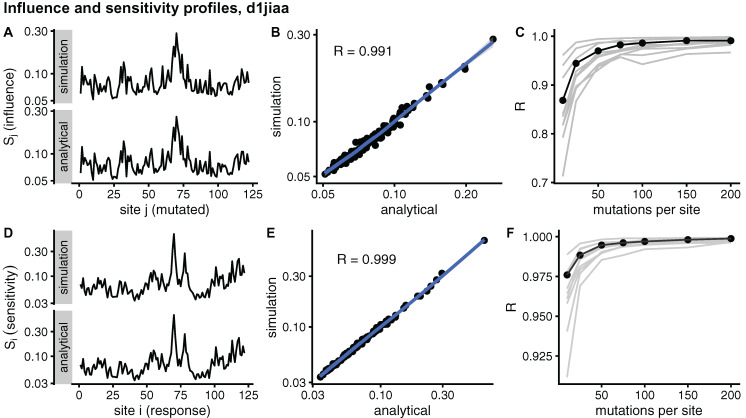
Comparison of sMRS and aMRS marginal profiles. Results shown for Phospholipase A2 (d1jiaa). The influence profile is the average of the sensitivity matrix over rows; element *S_j_* measures the average influence of mutations at site *j*. The sensitivity profile is the average of the response matrix over columns; element *S_i_* measures the average sensitivity of site *i*. (A) Sj profiles obtained with sMRS using 200 mutations per site (simulation) and aMRS (analytical); (B) scatter plot of the sMRS vs. aMRS *S_j_* values of A; (C) convergence of the sMRS *S_j_* profile towards the aMRS profile. (D) Si profiles obtained with sMRS using 200 mutations per site (simulation) and aMRS (analytical); (E) scatter plot of the sMRS vs. aMRS *S_i_* values of D; (F) convergence of the sMRS *S_i_* profiles towards the aMRS profile. In C and F, the d1jiaa case is shown with black lines and points, and the other 9 proteins studied are shown using grey lines. Profiles were calculated using the normalised matrix (matrix average is 1). Profile elements are shown in logarithmic scale and *R* is the Pearson correlation coefficient between log-transformed sMRS and aMRS profiles.

Figure 2 compares sMRS and aMRS profiles for Phospholipase A2 (d1jiaa) (Similar figures for the other proteins studied can be found in Supplemental_info.pdf). Comparing influence profiles, we see that sMRS with *M* = 200 and aMRS profiles are very similar (Fig. 2A and Fig. 2B) and that sMRS influence profiles converge rapidly towards the corresponding aMRS profiles as *M* increases (Fig. 2C). Similarly, the sensitivity profile estimated by sMRS with *M* = 200 is also very similar to its aMRS counterpart (Figs. 2D and 2E) and the sMRS profile converges rapidly towards the aMRS profile as *M* increases (Fig. 2F).

Similar results are found for the other proteins studied. The convergence of influence profiles (grey lines of Fig. 2C) is somewhat slower than that of sensitivity profiles (grey lines of Fig. 2F), but in both cases there is good convergence. For *M* = 200, Pearson’s correlation between sMRS and aMRS influence profiles is in the range 0.97 *≤ R ≤* 1.00 and the correlation between sensitivity profiles is 1.00 for all proteins (Table 2). In summary, sMRS influence and sensitivity profiles converge rapidly, so that with *M* = *O*(10^2^) they are very similar to their aMRS counterparts.

#### aMRS is much faster than sMRS

**Figure 3:**
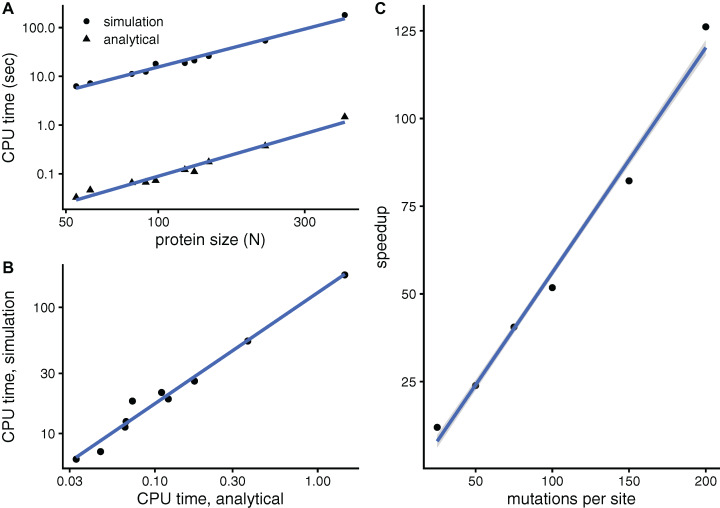
The analytical mutation-response scanning method (aMRS) is much faster than the simulation method (sMRS). (A) CPU time vs. protein size the for sMRS with 200 mutations per site (simulation) and for aMRS (analytical). Time is shown in logarithmic scale. From the slope of the linear fits it follows that both times scale with *N*^1.5^ (*N* is the number of sites, each point is one protein). (B) The CPU time of the simulation method increases linearly with the CPU time of the analytical method, with a speedup of 126: *t*_sMRS_ = 126×*t*_aMRS_. (C) The speedup, *t*_sMRS_/*t*_aMRS_ obtained as shown in B, increases linearly with the number of mutations per site. Calculations were performed on the proteins of Table 1 using the methods implemented in R, with base LAPACK and the optimised AtlasBLAS libraries for matrix operations, on an early-2018 MacBook Pro notebook (processor i7-8850H).

The purpose of this paper is to develop a faster mutation-response scanning method. To see whether aMRS is indeed faster than sMRS, Fig. 3 compares their computational cost. An sMRS calculation using a typical number of *M* = 200 mutations per site is much slower than an aMRS calculation (Fig. 3A). The computational cost, as measured by CPU time, scales with protein length as *N*^1.5^ for both sMRS and aMRS. As a result, *t*_sMRS_ increases linearly with *t*_aMRS_ with a slope that is the speedup of aMRS vs. sMRS; For the M = 200 case, this speedup is *t*_sDMRS_/*t*_aDMRS_ ≈ 126 (Fig. 3B). Further, the speedup increases linearly with *M*: *t*_sMRS_/*t*_aMRS_ ∝ *M* (Fig. 3C). Thus, the analytical method provides a speedup of the order of the number of mutations per site, which is typically in the hundreds. In a word, aMRS is much faster than sMRS.

### Double mutation-response scanning

This section assesses the analytical Double Mutation-Response Scanning method (aDMRS) by comparison with the simulation-based Double Mutation-Response Scanning method (sDMRS). These methods are alternative ways of calculating a compensation matrix **D**. This matrix is composed by elements *D*_*ij*_ that measure the degree to which mutating site *j* may compensate the structural deformation due to a first mutation at site *i* (Eq. (19)). The simulation method, sDMRS, obtains this matrix numerically scanning over pairs of simulated mutations (see “Simulation-based Double Mutation-Response Scanning”). The analytical method, aDMRS, calculates the compensation values using a closed formula (Eq. (26)), avoiding the use of simulations (see “Analytical Double Mutation-Response Scanning”).

#### sDMRS converges slowly towards aDMRS

I compare aDMRS with aDMRS for the proteins of Table 1. As in “Mutation-Response Scanning”, before addressing computational cost, I consider the convergence of the simulation method with increasing *M*.

**Figure 4:**
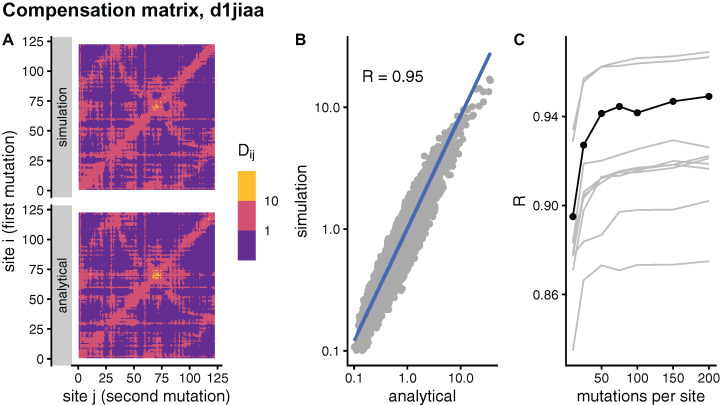
Comparison of sDMRS and aDMRS compensation matrices. Results shown for Phospholipase A2 (d1jiaa). The compensation matrix D has elements *D_ij_* that measure the maximum compensation of the structural deformation due to a mutation at site *i* afforded by a second mutation at *j*. sDMRS is a simulation-based Double Mutation Response Scanning method that calculates D by maximizing the structural compensation over pairs of simulated mutations. aDMRS is an analytical method that calculates D using a closed formula. (A) sDMRS compensation matrix obtained using 200 mutations per site (simulation) compared with the aDMRS matrix (analytical). (B) Scatterplot of the sDMRS vs. aDMRS matrix elements of A. (C) Convergence of the sDMRS matrix towards the aDMRS matrix with increasing number of mutations per site. In C the d1jiaa case is shown with black lines and points, and the other 9 proteins studied are shown with grey lines. *D_ij_* are normalised so that their average is 1, logarithmic scales are used in A and B, and *R* is Pearson’s correlation coefficient between log-transformed sDMRS and aDMRS matrix elements.

In principle, the simulation and analytical methods are equivalent. The compensation matrix **D** calculated with sDMRS with M→∞ will be identical to the aDMRS matrix. However, in practice the sDMRS matrix depends on *M*. Figure 4 compares simulated and analytical compensation matrices for the example case of Phospholipase A2 (SCOP id d1jiaa) (Similar figures for the other proteins studied can be found in Supplemental_info.pdf). First, note that the compensation matrix obtained with sDMRS with *M* = 200 looks similar to the aDMRS matrix (Fig. 4A). More quantitatively, a scatter plot of sDMRS vs. aDMRS matrix elements shows good correlation, but there is a visible scattering of points around the linear fit (Fig. 4C). The similarity between sDMRS and aDMRS matrices can be measured by the correlation coefficient, which in this case is *R* = 0.95. Figure 4C shows that as *M* increases, the sDMRS matrix converges rapidly at first towards the aDMRS matrix, but convergence slows down with further increases of *M*. Thus, for Phospholipase A2, sDMRS with *O*(10^2^) mutations per site produces a compensation matrix that is in good agreement with, but not identical to, the aDMRS matrix.

A similar situation is found for the other proteins of the dataset. Convergence quickly slows down as *M* increases (see grey lines of Fig. 4C ). For *M* = 200, the correlation between sDMRS and aDMRS matrices falls within the range 0.87 *≤ R ≤* 0.97 (Table 3). Thus, the sDMRS compensation matrix converges slowly towards the aDMRS matrix, so that for *M* = *O*(10^2^) the simulated matrix is in moderate to good agreement with the analytical matrix. The degree of convergence is not clearly related to protein properties such as structural class or protein size, thus convergence should be tested whenever the simulation method is used.

**Table 3:** aDMRS vs. sDMRS summary.

protein	*N*	*t*_sDMRS_	*t*_aDMRS_	*R*	*R*_*i*_	*R*_*j*_
d1lcka1	54	22.24	0.21	0.87	0.76	0.78
d1ntxa	60	27.70	0.17	0.97	0.97	0.99
d1fxla2	82	59.58	0.43	0.93	0.89	0.94
d1bxva	91	77.46	0.60	0.92	0.55	0.69
d2acya	98	116.38	0.76	0.90	0.56	0.71
d1jiaa	122	167.89	1.24	0.95	0.85	0.94
d1hmta	131	203.65	1.30	0.97	0.77	0.93
d1a4fb	146	274.29	2.13	0.92	0.70	0.83
d1mcta	223	1,034.36	11.95	0.92	0.64	0.74
d2l8ma	405	12,995.91	56.53	0.92	0.61	0.77

**Note:**

N: protein length; *t*_sDMRS_: CPU time of sDMRS in seconds; *t*_aDMRS_: CPU time of aDMRS in seconds. Convergence measures at *M* = 200 mutations per site: *R*: correlation coefficient between sDMRS and aDMRS compensation matrices **D**; *R*_*i*_: correlation between *D*_*i*_ profiles; *R*_*j*_: correlation between *D*_*j*_ profiles.

**Figure 5:**
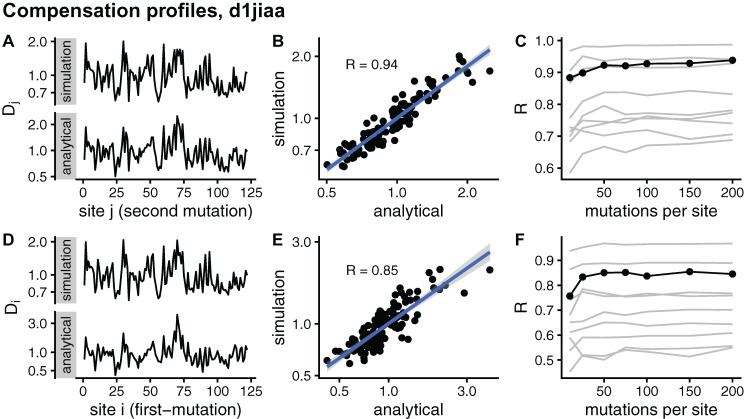
Comparison of sDMRS and aDMRS marginal profiles. Results shown for Phospholipase A2 (d1jiaa). Two marginal profiles are considered. The *D_j_* profile is the average of the compensation matrix over rows; element *D_j_* measures the ability of *j* to compensate mutations at other sites. The *D_i_* profile is the average of the compensation matrix over columns; element *D_i_* measures the degree to which a mutation at *i* can be compensated by mutations elsewhere. (A) sDMRS *D_j_* profile obtained using 200 mutations per site (simulation) and aDMRS *D_j_* profile (analytical); (B) scatter plot of the sDMRS vs. aDMRS *D_j_* values of A; (C) convergence of the sDMRS *D_j_* profile towards the aDMRS profile. (D) sDMRS *D_i_* profile obtained using 200 mutations per site (simulation) and aDMRS *D_i_* profile (analytical); (E) scatter plot of the sDMRS vs. aDMRS *D_i_* values of D; (F) convergence of the sDMRS *D_i_* profile towards the aDMRS profile. In C and F, the d1jiaa case is shown with black lines and points, and the other 9 proteins studied are shown with grey lines. Profiles were calculated with normalised matrices (matrix average is 1), they are in logarithmic scale, and *R* is the Pearson correlation coefficient between the log-transformed sDMRS and aDMRS profiles.

I further assess convergence by considering site-dependent compensation profiles. Averaging **D** over rows, I obtain a *D*_*j*_ profile that measures the average compensation power of sites *j*. Averaging over columns, I obtain a *D*_*i*_ profile that measures how likely to be compensated mutations at *i* are. Figure 5 compares sDMRS and aDMRS profiles for Phospholipase A2 (d1jiaa). The *M* = 200 sDMRS profiles are visually similar to aDMRS profiles (Fig. 5A and Fig. 5D). The similarity is not very high, however: points are quite scattered around the linear fit in sDMRS vs. aDMRS plots (Fig. 5B and Fig. 5E). The convergence of sDMRS profiles towards their aDMRS counterparts is very slow (Fig. 5C and Fig. 5F).

Similar results are found for the other proteins studied. Profiles generally improve very slowly with increasing *M* (see grey lines of Fig. 5C and Fig. 5F). For *M* = 200, the correlation coefficient between sDMRS and aDMRS *D*_*i*_ profiles falls in the range 0.55 *≤ R ≤* 0.97 and between *D*_*j*_ profiles it falls in the range 0.69 *≤ R ≤* 0.99 (Table 3). In summary, sDMRS profiles converge very slowly with increasing *M*, so that for *M* = *O*(10^2^), they are often poorly converged. In addition, There are no obvious determinants of convergence: *R* is not clearly determined by either protein size or structural class. Therefore, whenever the simulation method is used, convergence should be tested.

#### aDMRS is much faster than sDMRS

**Figure 6:**
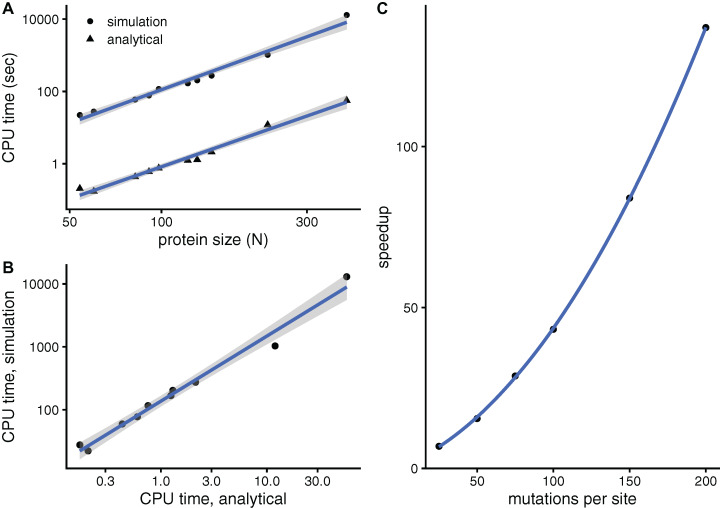
The analytical double mutation-response scanning method (aDMRS) is much faster than the simulation method (aDMRS). (A) CPU time vs. protein size for sDMRS with 200 mutations per site (simulation) and for aDMRS (analytical). Time is shown in logarithmic scale. From the slope of the linear fits it follows that both CPU times scale with *N*^3^ (*N* is the number of sites, each point is one protein). (B) The CPU time of the simulation method increases linearly with the CPU time of the analytical method, with a speedup of 137: *t*_sDMRS_ = 137×*t*_aDMRS_. (C) The speedup, *t*_sDMRS_/*t*_aDMRS_, increases non-linearly with the number of mutations per site *M*, tending towards *O*(*M*^2^) for large *M*. Calculations were performed on the proteins of Table 1 using the methods implemented in R, with base LAPACK and the optimised AtlasBLAS libraries for matrix operations, on an early-2018 MacBook Pro notebook (processor i7-8850H).

To see whether aDMRS is faster than aDMRS, Fig. 6 compares their computational cost. sDMRS with *M* = 200 mutations per site is much slower than aDMRS (Fig. 6A). The computational cost, as measured by CPU time, scales with protein length as *N*^3^ for both sDMRS and aDMRS. As a result, *t*_sDMRS_ increases linearly with *t*_aDMRS_ with a slope that is the speedup of aDMRS vs. sDMRS. For the *M* = 200 case, *t*_sDMRS_/*t*_aDMRS_ ≈ 137 (Fig. 6B). The speedup increases non-linearly with *M* (Fig. 6C). This dependence can be understood from the sDMRS procedure schematised in “Simulation-based Double Mutation-Response Scanning”. The cost of generating the mutations (steps 3 and 4) increases linearly with *M*, while performing the average and maximization needed to calculate the compensation matrix (steps 5) scales as *M*^2^. Therefore, for large *M* the analytical method provides a speedup of *O*(*M*^2^), making aDMRS much faster than sDMRS.

## Discussion

I have derived, implemented, and assessed two mutation-response scanning methods, aMRS and aDMRS, which are analytical alternatives to the simulation methods sMRS and sDMRS, respectively. All methods were implemented using R with optimized BLAS and LAPACK libraries. None of the methods posed major implementation difficulties.

The methods were assessed on a dataset of 10 proteins of varying lengths. First, I consider the convergence of simulation methods. In the limit if infinite mutations per site (*M*), simulation and analytical methods should give the same results. In practice, the degree of convergence of the simulation methods depends on *M*. sMRS converges rapidly towards aMRS, so that with a typical *M* = *O*(10^2^) the sDMRS sensitivity matrix and its marginal profiles are almost identical to those calculated with aMRS (Fig. 1C, Fig. 2C, Fig. 2F, Table 2). On the other hand, sDMRS converges slowly, so that even with *M* = *O*(10^2^) sDMRS convergence is not guaranteed (Fig. 4C, Fig. 5C, Fig. 5F, Table 3). sDMRS converges more slowly than sMRS because it is more difficult to find extreme values (calculation of the compensation matrix involves maximization over pairs of mutations) than averages (sensitivity matrix elements are averages over mutations). In general, when using simulation-based methods convergence should always be assessed. In contrast, since the analytical methods do not depend on *M*, there is no need to study convergence, and possible convergence issues are altogether avoided.

Beyond convergence, since the purpose of this work was to develop faster methods, the key finding is that the analytical methods are much faster than the simulation methods. For a typical case of *M* = 200 mutations per site, aMRS is 126 × faster than sMRS and aDMRS is 137 × faster than sDMRS. While the computational cost of sMRS is relatively modest and increases rather slowly in proportion to *N*^1.5^ *M*, sDMRS is much more computationally expensive and its cost rises steeply in proportion to *N*^3^*M*^2^. The speedup of analytical methods is of *O*(*M*) for single-mutation scans and *O*(*M*^2^) for double-mutation scans. This speedup may be most important for large proteins. For instance, for the 405-sites-long Cytochrome P450, an sMRS calculation takes 3 CPU min vs. 1.5 s of the alternative aMRS calculation (Table 2). On the other hand, an sDMRS calculation takes 3.6 h vs. 1 min of the alternative aDMRS calculation (Table 3). Therefore, there is a large speedup for both single and double mutation-response scans, that may be most useful for the later case.

To further compare the mutation-response scanning methods considered here, I discuss some of their main limitations. All methods are based on the Linear Response Approximation formula Δ**r**^0^ = **Cf**. Therefore, the main limitations are the validity of LRA, the quality of **C**, and how well mutations can be modelled by the force **f**. Regarding the first limitation, LRA will be valid if both perturbations (**f**) and their responses (Δ**r**^0^) are small. Thus LRA should be valid for most mutations, failing only in the rare cases in which specific mutations induce very large conformational changes. Second, calculating **C** with a simple elastic network model, as done here, might impose additional limitations. However, this could be alleviated by calculating **C** using more sophisticated means, such as MD simulations, if necessary. More fundamentally, the main limitation is the very assumption that **C** characterizes the conformational ensemble, which will be the case for proteins with a single native structure, but may fail for proteins that have two or more stable conformations. The final limitation depends on whether mutations can be adequately modelled using forces (**f**). While it is possible that this fails for the prediction of specific mutations, mutations-as-forces models have been proved successful in many previous studies that depend on summary statistics such averages or maxima (Echave, 2008; Echave & Fernández, 2010; Tiberti et al., 2018; Marcos & Echave, 2020). For the present work, it should be noted that the limitations mentioned are common to the simulation methods and their analytical alternatives. The analytical approach adds no limitation to the list.

Given that limitations exist, it is worthwhile to discuss why this work has not validated the methods by comparison with empirical data. The main reason is that the aim of this work is not to develop mutation-response methods in better agreement with experiment, but to develop faster methods. This is why the assessment was performed by comparing between simulation and analytical approaches, rather than validating such approaches against empirical data. Validating mutation-response scanning itself is beyond the scope of this work. A second reason is that taking the validity of mutation-response scanning as a given is reasonable. For 8 of the proteins of Table 1, the mutation-response model of the present paper has been recently validated by comparison with empirical structural sensitivity profiles Marcos & Echave (2020). More generally, the validity of perturbation-response methods follows from their extensive successful use in a variety of applications for at least 15 years, as mentioned in “Introduction”.

The main conclusion of this work is that the analytical methods should be chosen over the simulation methods because they are faster and, in addition, they have no convergence issues. Therefore, the analytical methods should be useful for a wide range of potential applications, such as predicting evolutionary divergence of protein structures (Echave & Fernández, 2010; Marcos & Echave, 2020), detecting and interpreting pathological mutations (Nevin Gerek, Kumar & Banu Ozkan, 2013; Raimondi et al., 2018; Verkhivker, 2019), and detecting compensating mutations and rescue sites (Tiberti et al., 2018). The speedup afforded by the analytical methods would be especially helpful for treating otherwise intractable large proteins, protein complexes, and large protein databases.

To finish, I mention two possible lines of further development. A first line is to derive analytical expressions for the deformations caused by external forces applied to single sites, as in Perturbation-Response Scanning (PRS) (Atilgan & Atilgan, 2009; General et al., 2014) and Double Force Scanning (DFS) (Tiberti et al., 2018). This will be useful for applications related to ligand-binding induced deformations (Atilgan et al., 2010; General et al., 2014). Beyond deformations, a second line of development is to derive analytical alternatives to simulation-based methods that calculate effects of mutations on protein motions (Hamacher, 2008; Zheng & Tekpinar, 2009; Zheng & Thirumalai, 2009; Echave, 2012; Hamacher, 2008). This would be important for studies of the role of protein dynamics in function and evolution (Echave, 2012; Micheletti, 2013; Ponzoni & Bahar, 2018; Zhang et al., 2019; Zhang & Su, 2019; Wingert et al., 2021).

## Supplemental Information

10.7717/peerj.11330/supp-1 Supplemental Information 1 Supplementary information.

## Additional Information and Declarations

### Data Availability

The following information was supplied regarding data availability: The data and code are available at Zenodo. Data: Echave, Julian. (2020). Fast and exact single and double mutation-response scanning of proteins: data and results (Version 1.0.0) [Data set]. Zenodo. DOI 10.5281/zenodo.4122623. Code: Echave, Julian. (2020, October 23). Fast and exact single and double mutation-response scanning of proteins: code (Version 1.0.0). Zenodo. DOI 10.5281/zenodo.4123149.
