# Supplementary material for: Fast computational mutation-response scanning of proteins
Source: PeerJ. 2021 Apr 21;9:e11330. doi: 10.7717/peerj.11330 (PMC8067912; doi:10.7717/peerj.11330)

Fast computational mutation-response scanning of proteins
Supplemenatary figures

Julian Echave*

Instituto de Ciencias Físicas, Escuela de Ciencia y Tecnología, Universidad Nacional de San Martín, Martín de Irigoyen 3100, 1650 San Martín, Buenos Aires, Argentina

E-mail: jechave@unsam.edu.ar

The following pages contains figures similar to those of the main document for the other proteins of the dataset studied. See captions of main figures for details.

Sensitivity matrix, d1lcka1

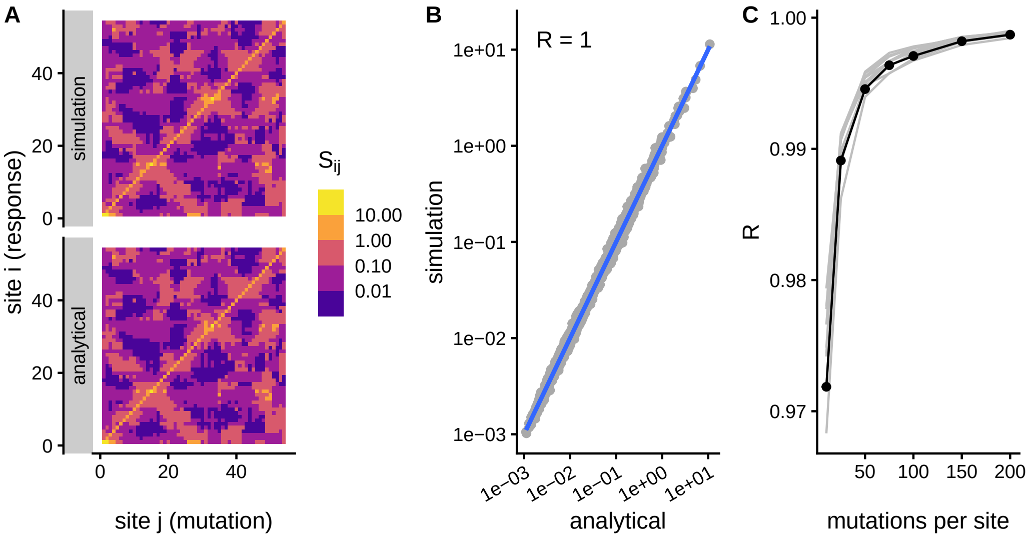

Influence and sensitivity profiles, d1lcka1

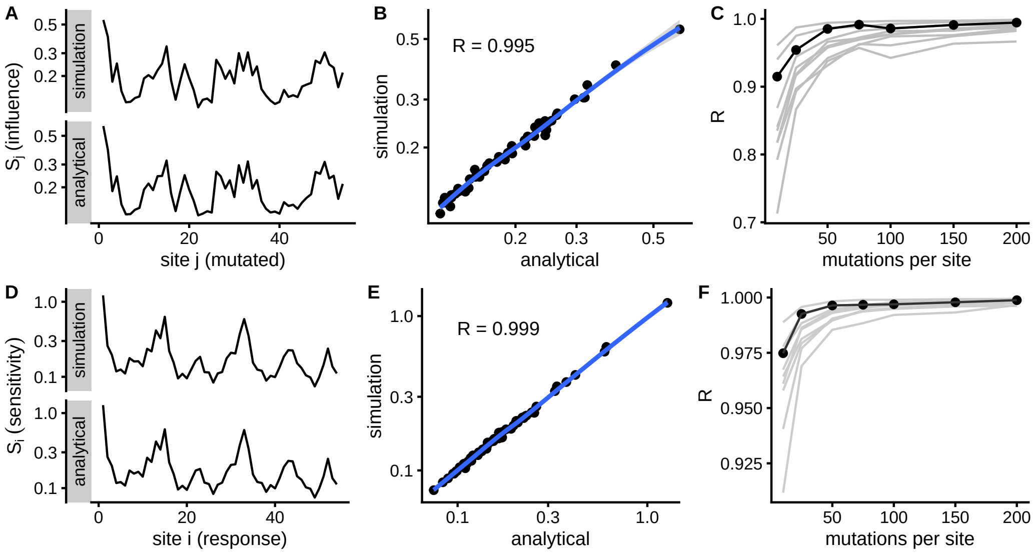

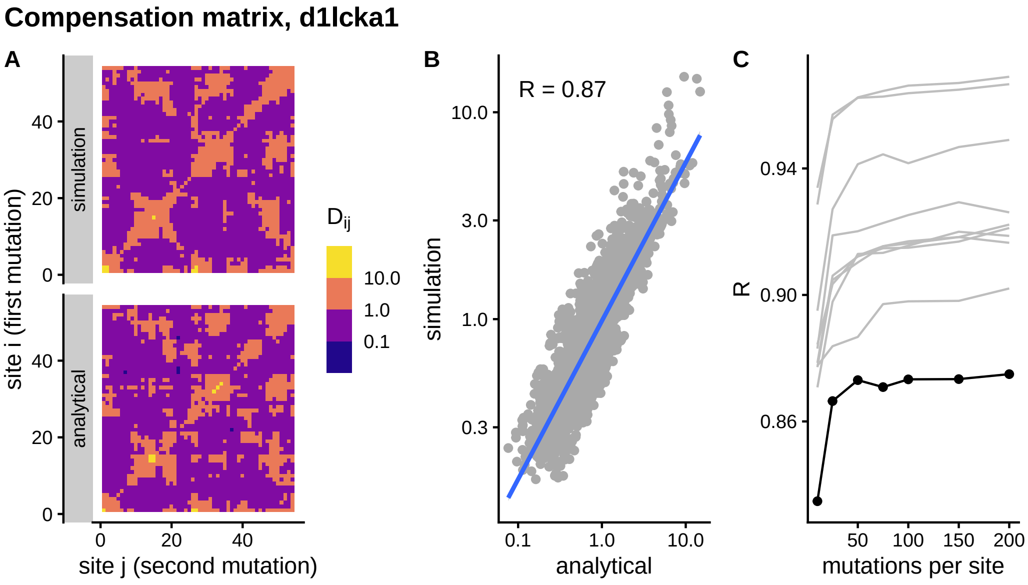
Compensation matrix, d1lcka1
A
B
C
simulation
analytical
site i (first mutation)
site j (second mutation)
D_{ij}
10.0
1.0
0.1
R = 0.87
simulation
analytical
R
mutations per site

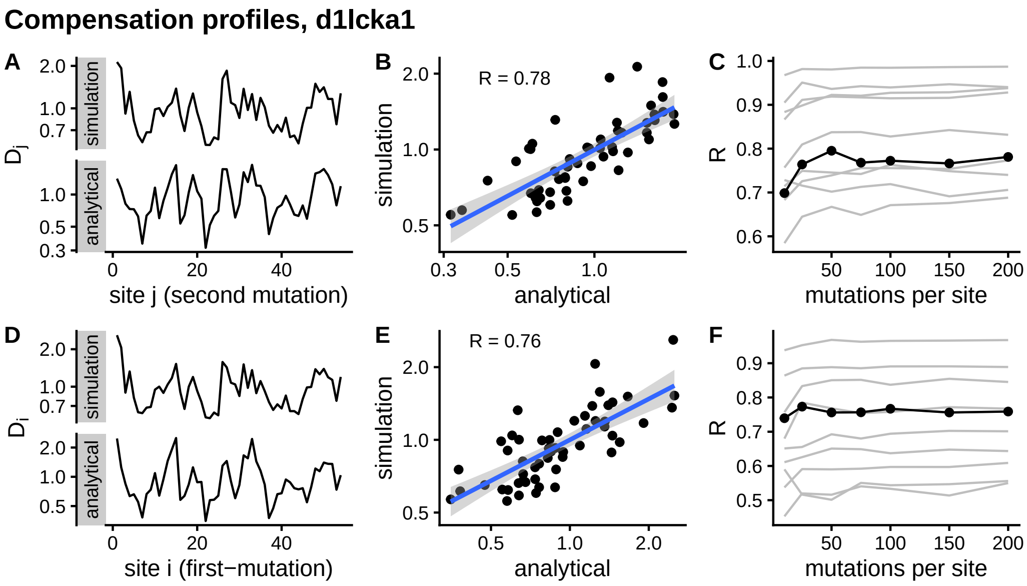
Compensation profiles, d1lcka1
A
B
C
D
E
F
D_j
D_i
simulation
analytical
site j (second mutation)
site i (first−mutation)
R = 0.78
R = 0.76
R
mutations per site

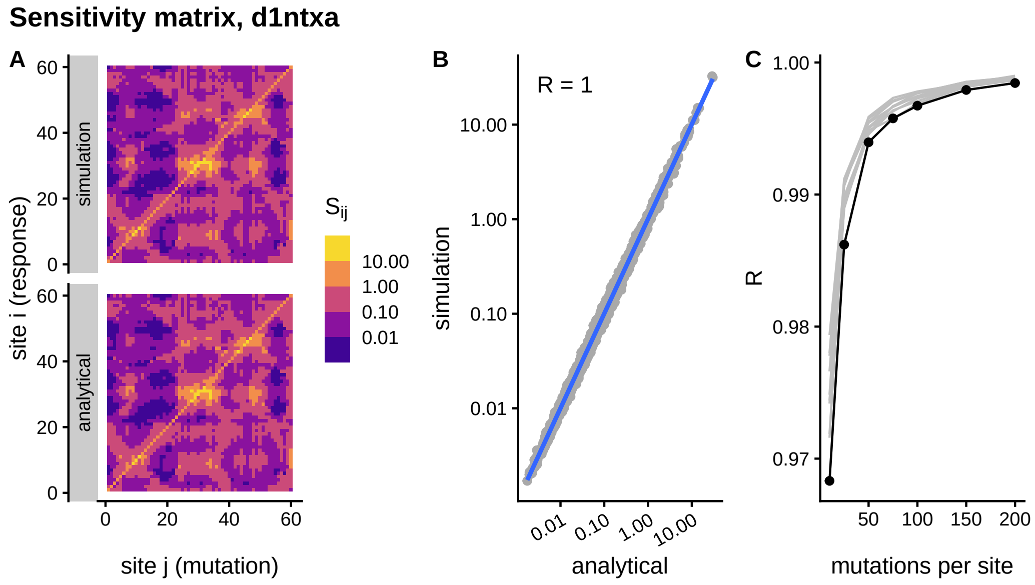

Sensitivity matrix, d1ntxa
A
B
C
site i (response)
simulation
analytical
site j (mutation)
S_{ij}
10.00
1.00
0.10
0.01
R = 1
simulation
analytical
R
mutations per site

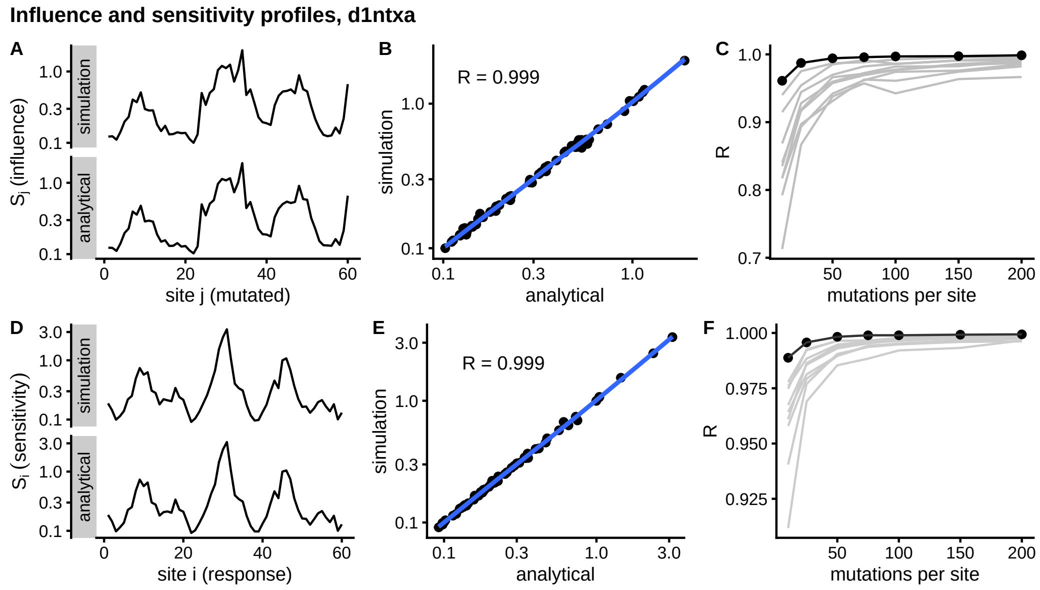

Influence and sensitivity profiles, d1ntxa
A
B
C
D
E
F
S_j (influence)
site j (mutated)
R = 0.999
simulation
analytical
R
mutations per site
S_i (sensitivity)
site i (response)
R = 0.999

Compensation matrix, d1ntxa

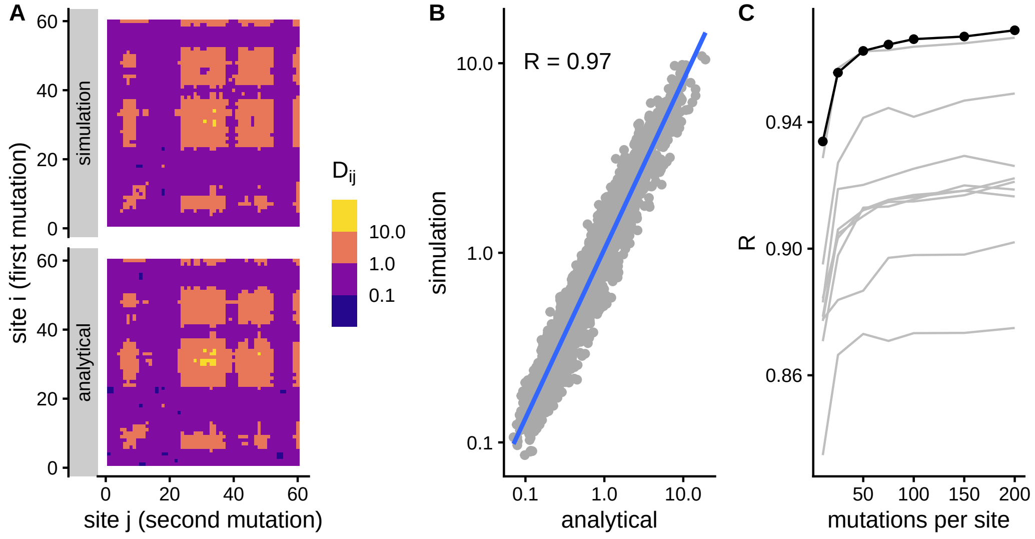

Compensation profiles, d1ntxa

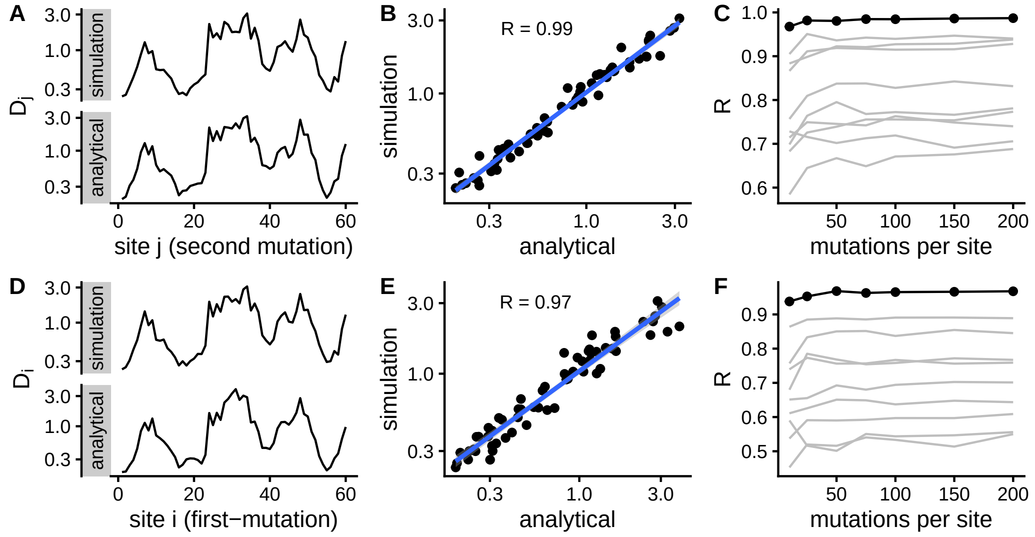

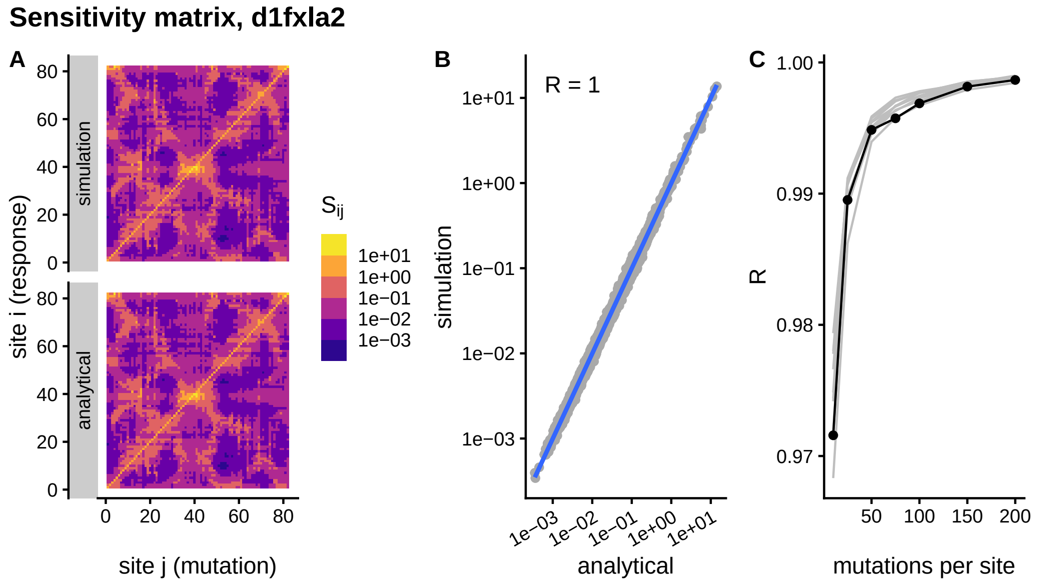
Sensitivity matrix, d1fxla2
A
B
C
site i (response)
simulation
analytical
site j (mutation)
S_{ij}
1e+01
1e+00
1e−01
1e−02
1e−03
R = 1
simulation
analytical
R
mutations per site

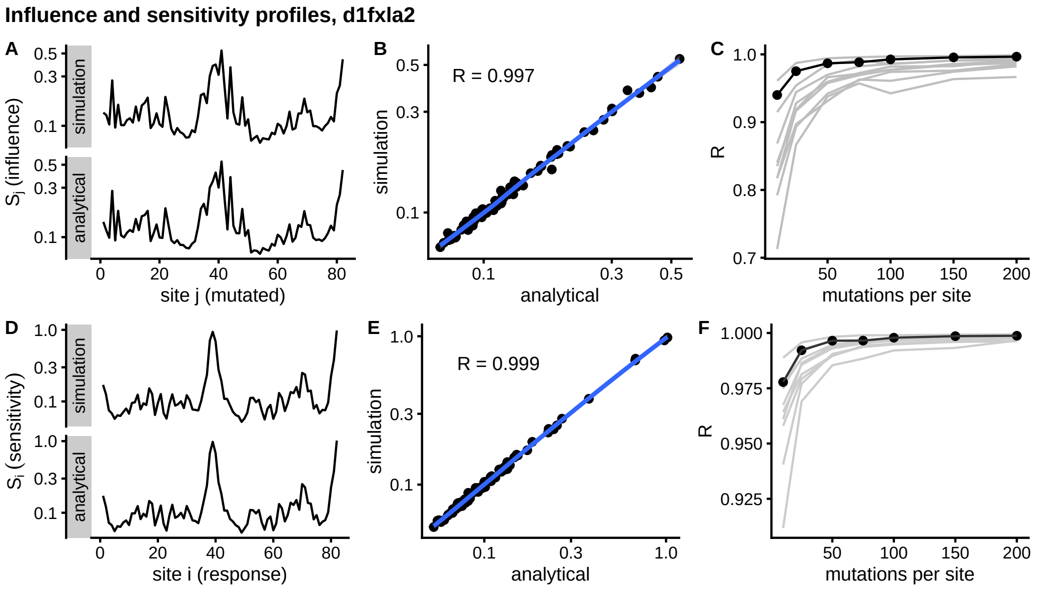
Influence and sensitivity profiles, d1fxla2
A
B
C
D
E
F
S_j (influence)
simulation
analytical
site j (mutated)
R = 0.997
simulation
analytical
R
mutations per site
S_i (sensitivity)
site i (response)
R = 0.999

Compensation matrix, d1fxla2

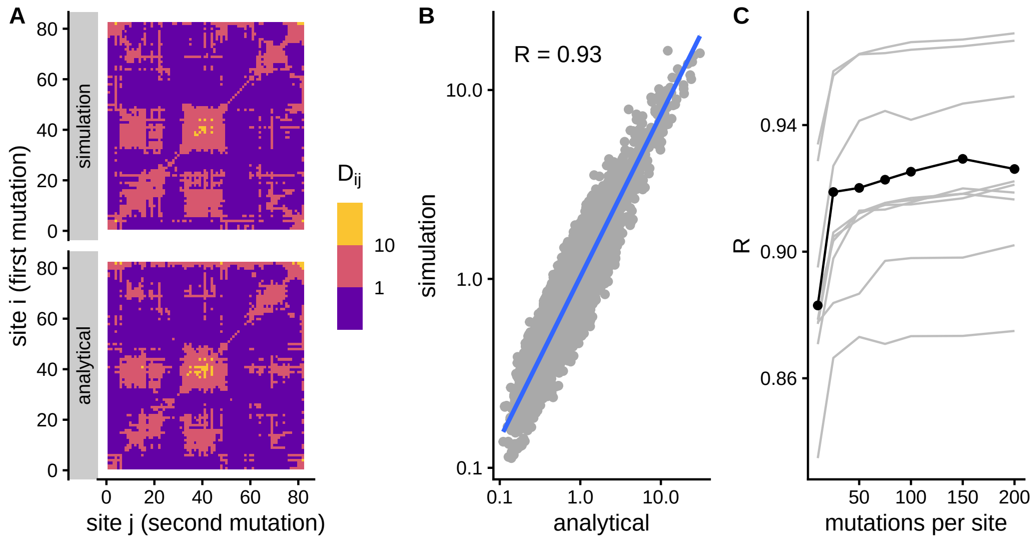

Compensation profiles, d1fxla2

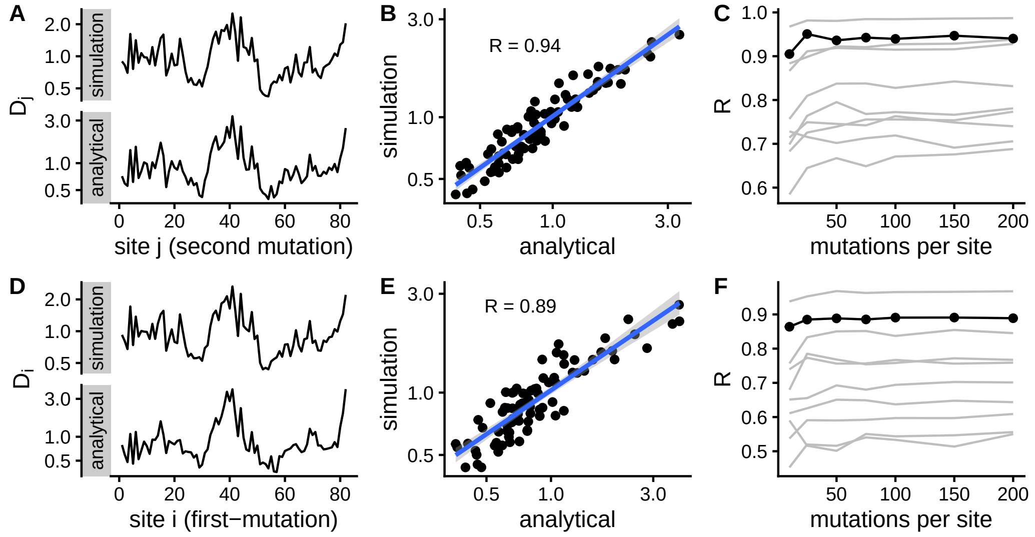

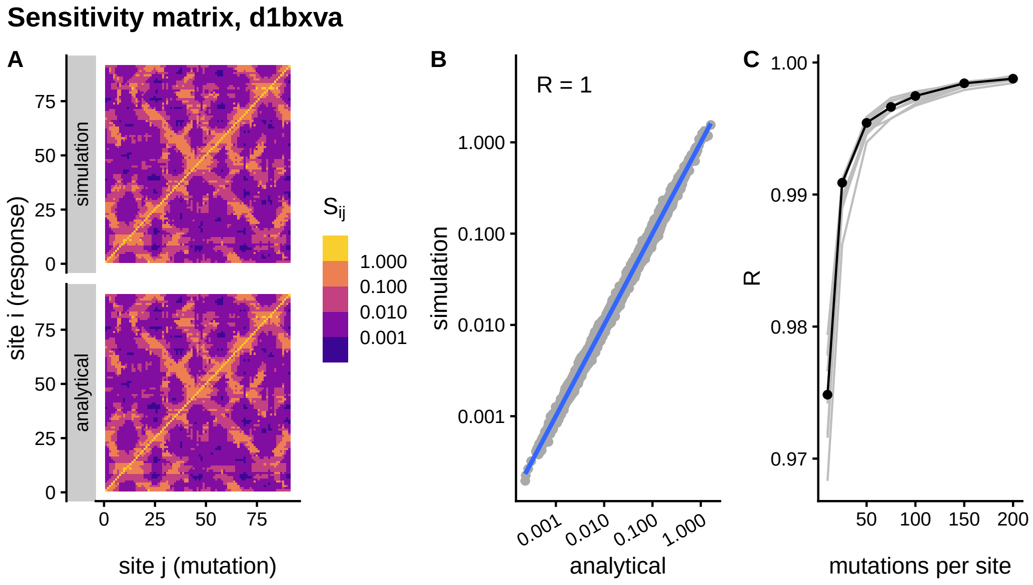
Sensitivity matrix, d1bxva
A
B
C
site i (response)
simulation
analytical
site j (mutation)
S_{ij}
1.000
0.100
0.010
0.001
R = 1
simulation
analytical
R
mutations per site

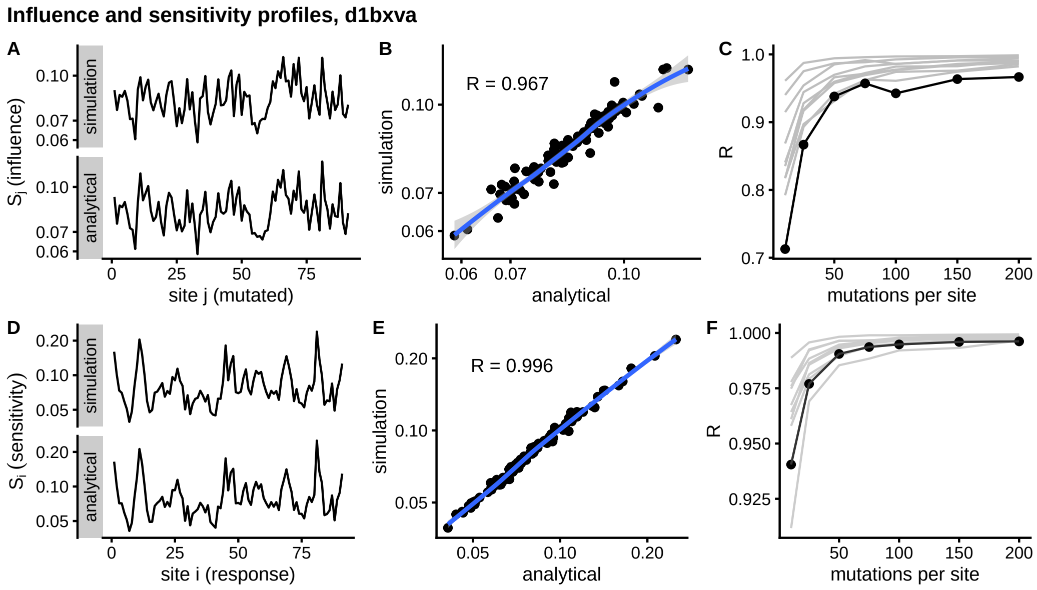
Influence and sensitivity profiles, d1bxva
A
B
C
D
E
F
S_j (influence)
simulation
analytical
site j (mutated)
R = 0.967
R = 0.996
R
mutations per site
S_i (sensitivity)
site i (response)

Compensation matrix, d1bxva

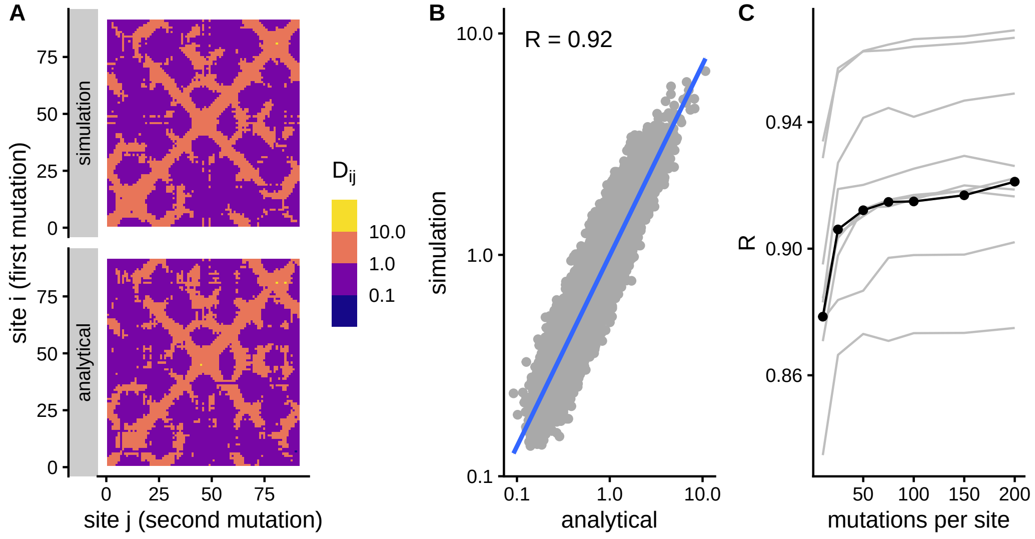

Compensation profiles, d1bxva

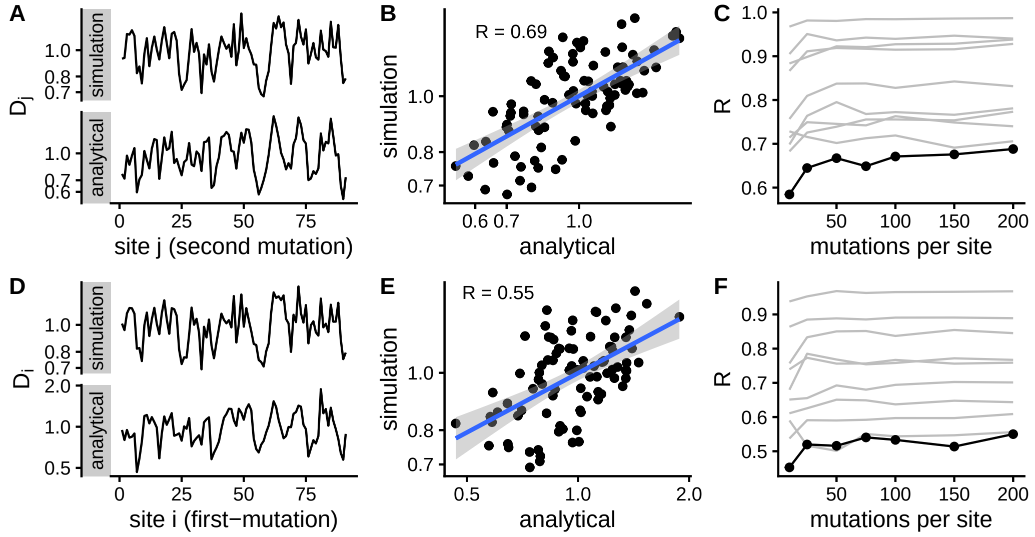

Sensitivity matrix, d2acya

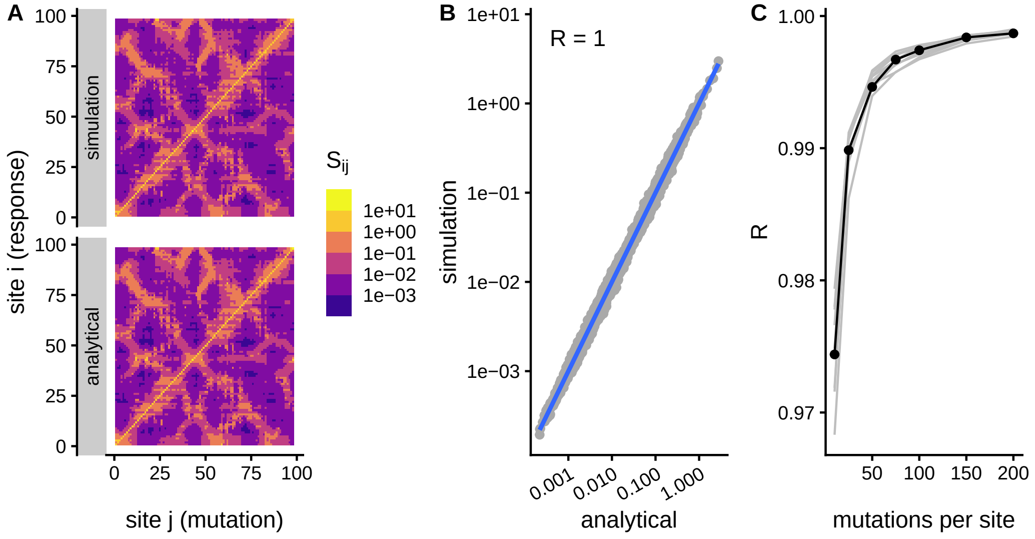

Influence and sensitivity profiles, d2acya

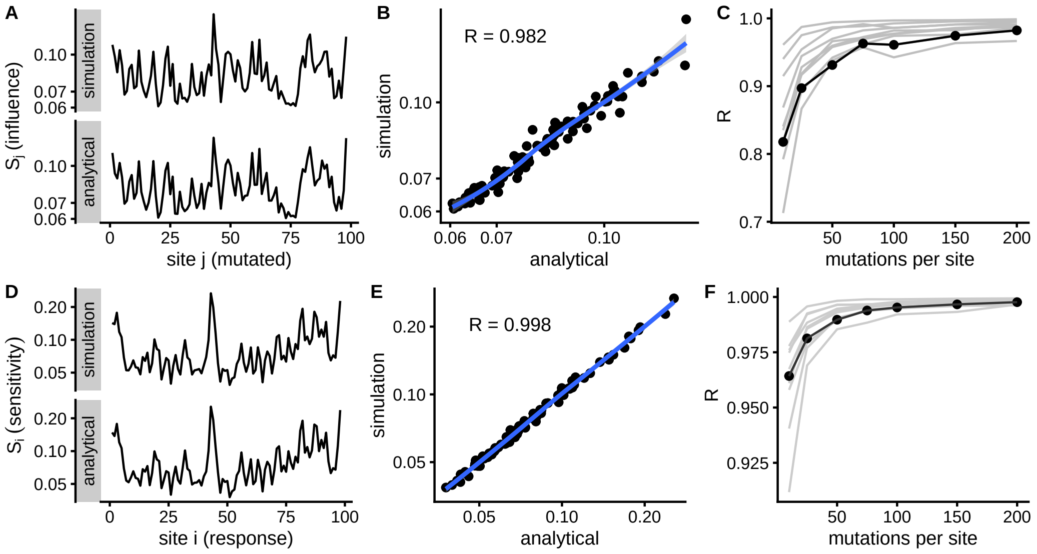

Compensation matrix, d2acya

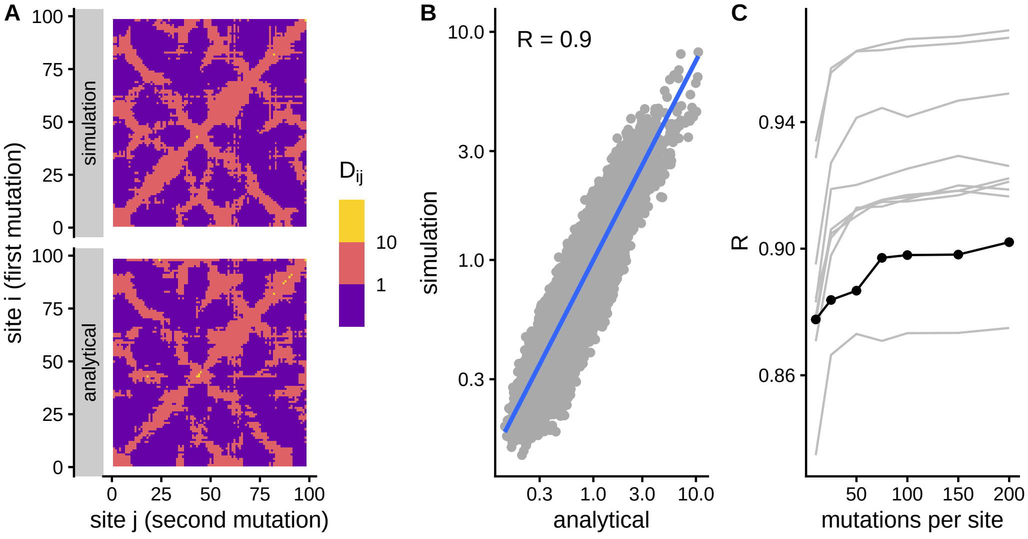

Compensation profiles, d2acya

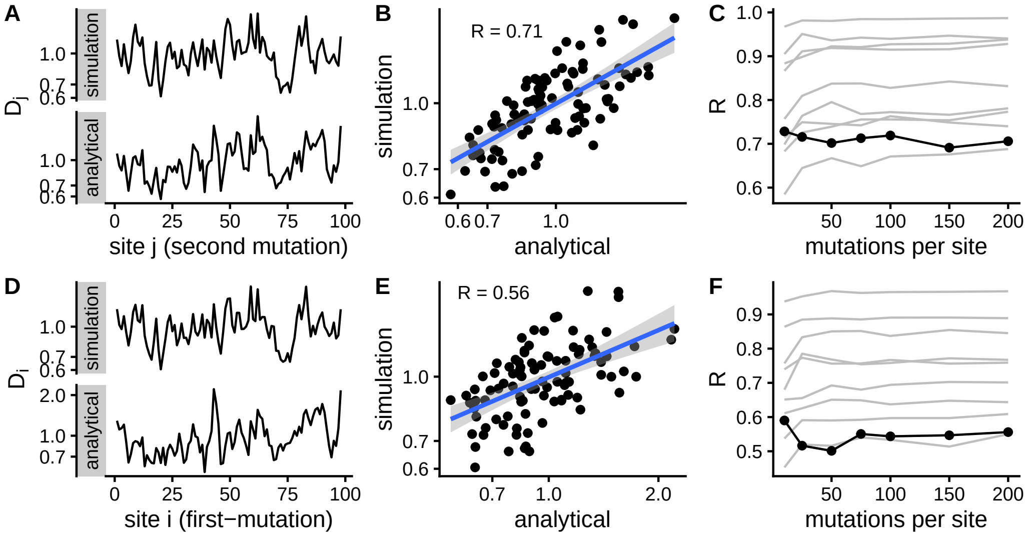

Sensitivity matrix, d1hmta

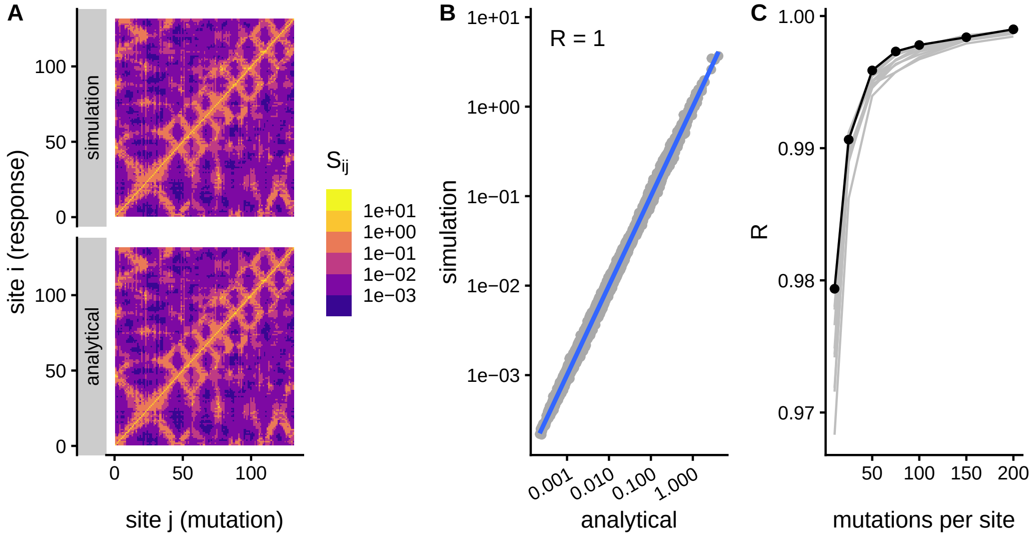

Influence and sensitivity profiles, d1hmta

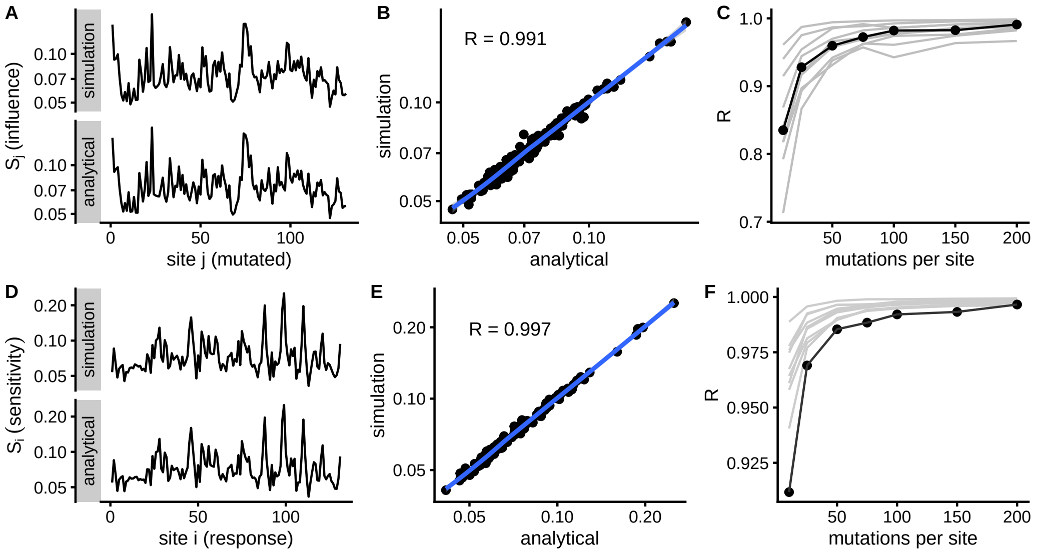

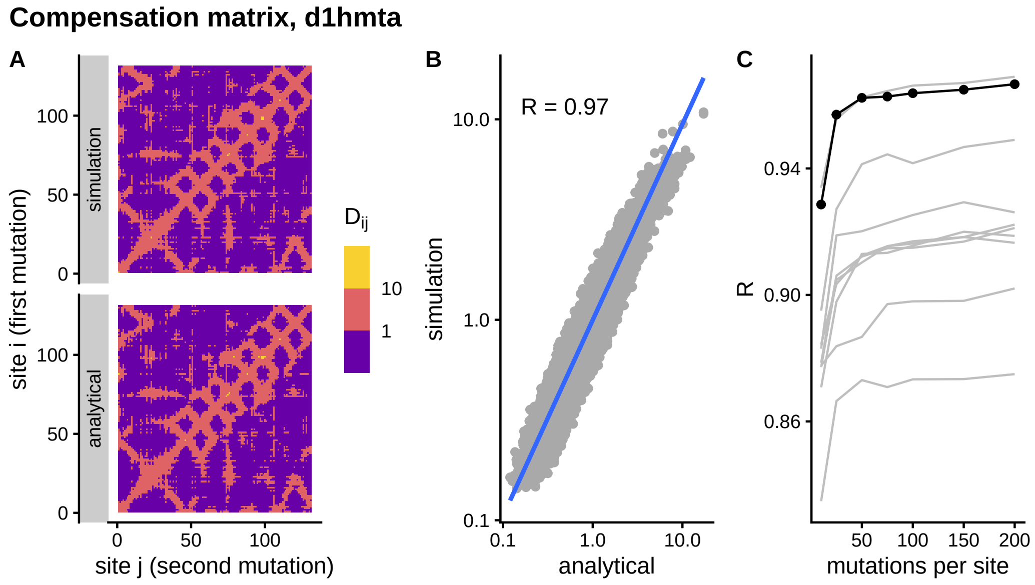
Compensation matrix, d1hmta
A
B
C
simulation
analytical
site i (first mutation)
site j (second mutation)
Dij
10
1
R = 0.97
simulation
analytical
R
mutations per site

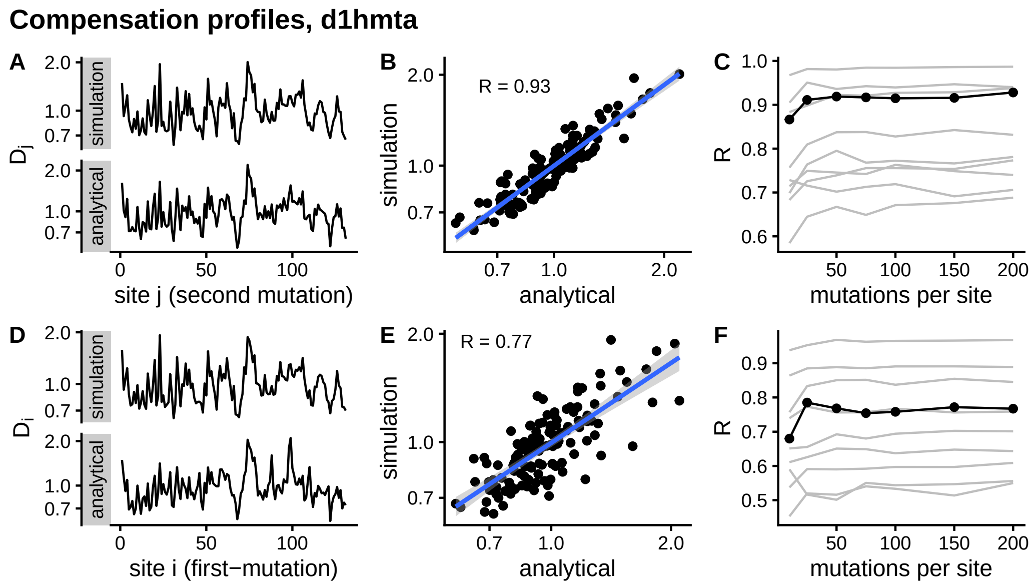
Compensation profiles, d1hmta
A
B
C
D
E
F
simulation
analytical
Dj
Di
site j (second mutation)
site i (first−mutation)
R = 0.93
R = 0.77
R
mutations per site

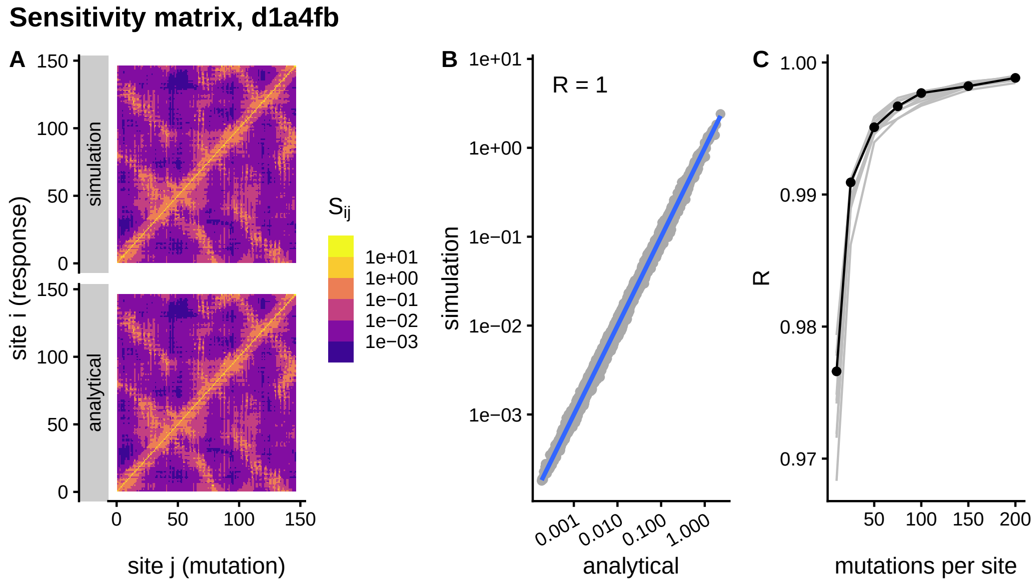

Sensitivity matrix, d1a4fb
A
B
C
site i (response)
simulation
analytical
site j (mutation)
S_{ij}
1e+01
1e+00
1e−01
1e−02
1e−03
R = 1
simulation
analytical
R
mutations per site

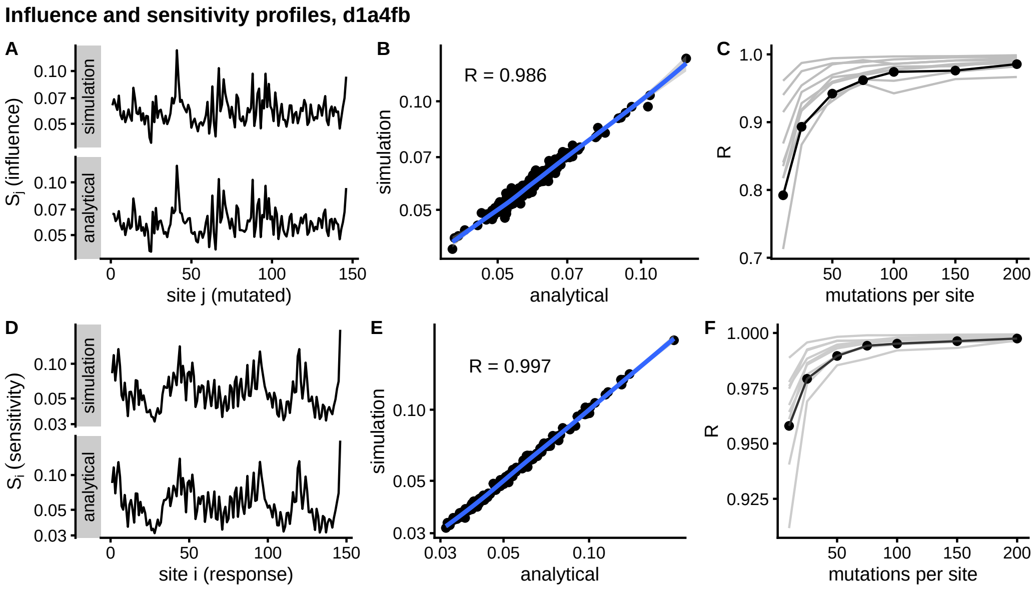

Influence and sensitivity profiles, d1a4fb
A
B
C
D
E
F
S_j (influence)
simulation
analytical
site j (mutated)
R = 0.986
simulation
analytical
R
mutations per site
S_i (sensitivity)
site i (response)
R = 0.997

Compensation matrix, d1a4fb

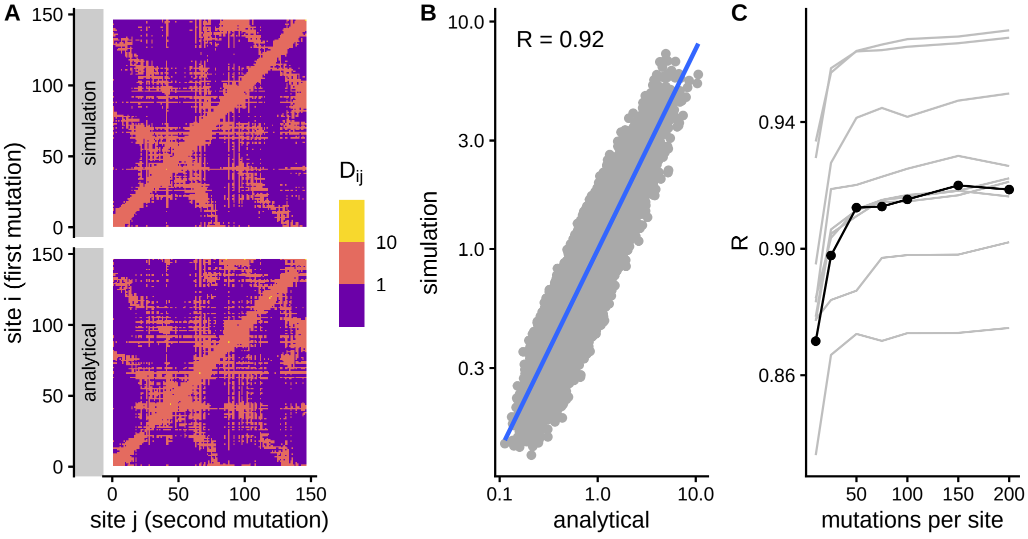

Compensation profiles, d1a4fb

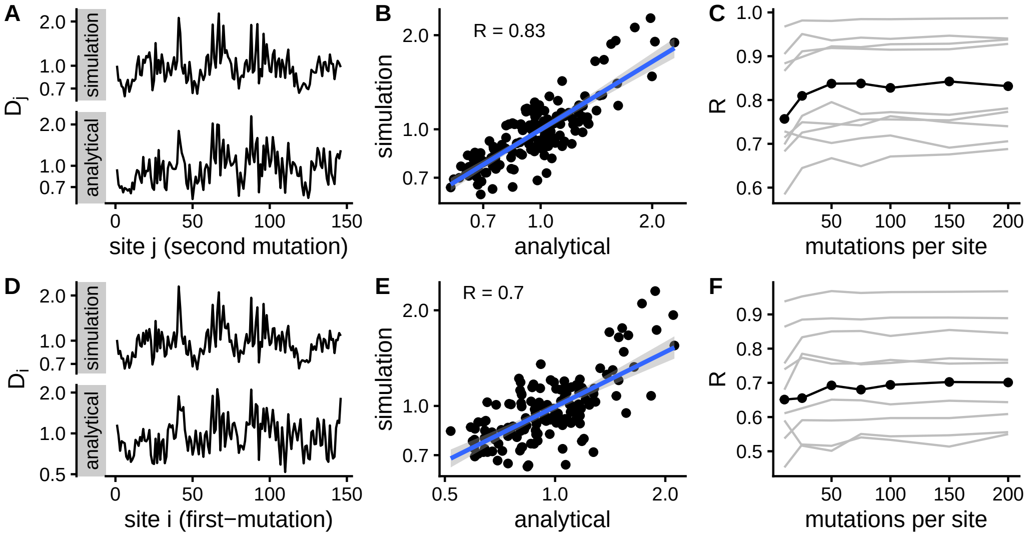

Sensitivity matrix, d1mcta

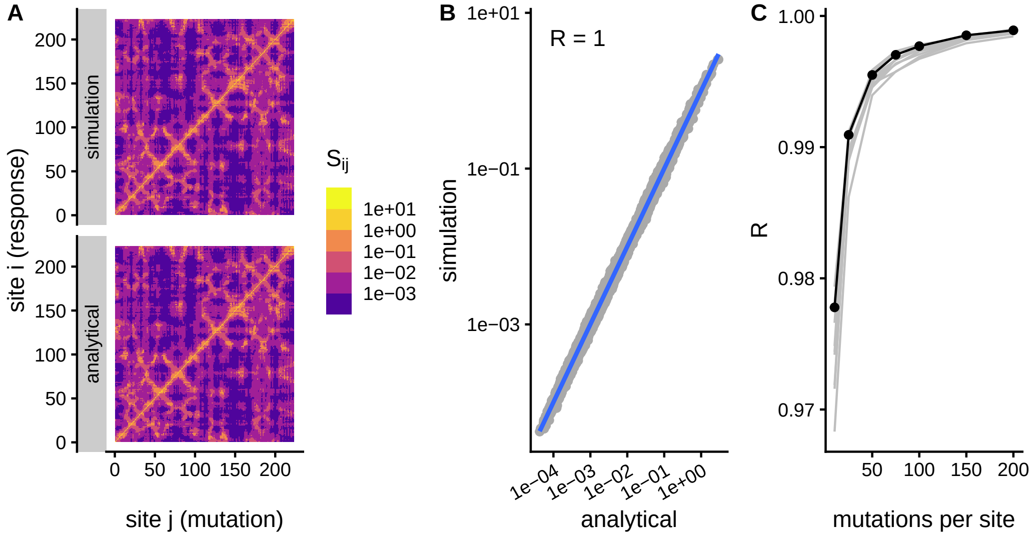

Influence and sensitivity profiles, d1mcta

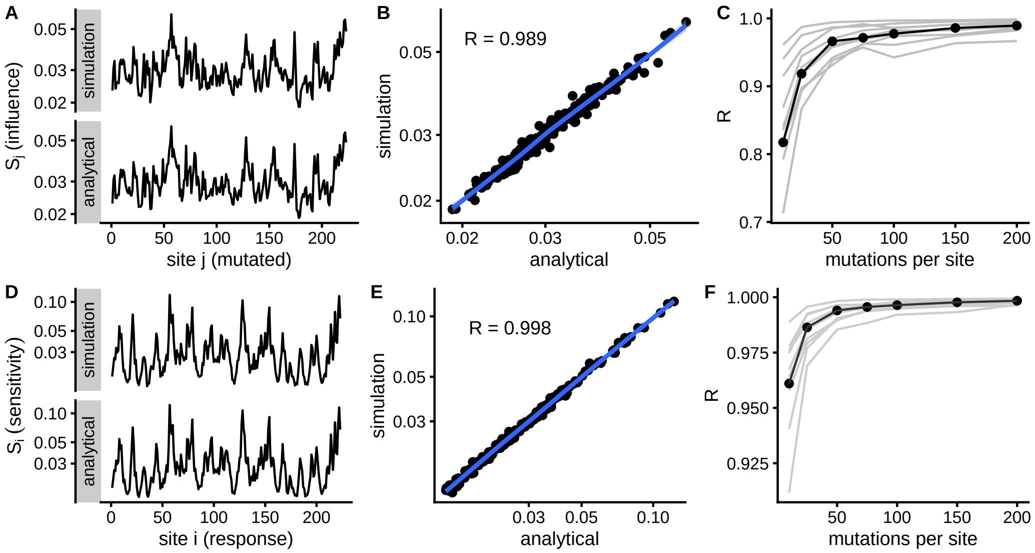

Compensation matrix, d1mcta

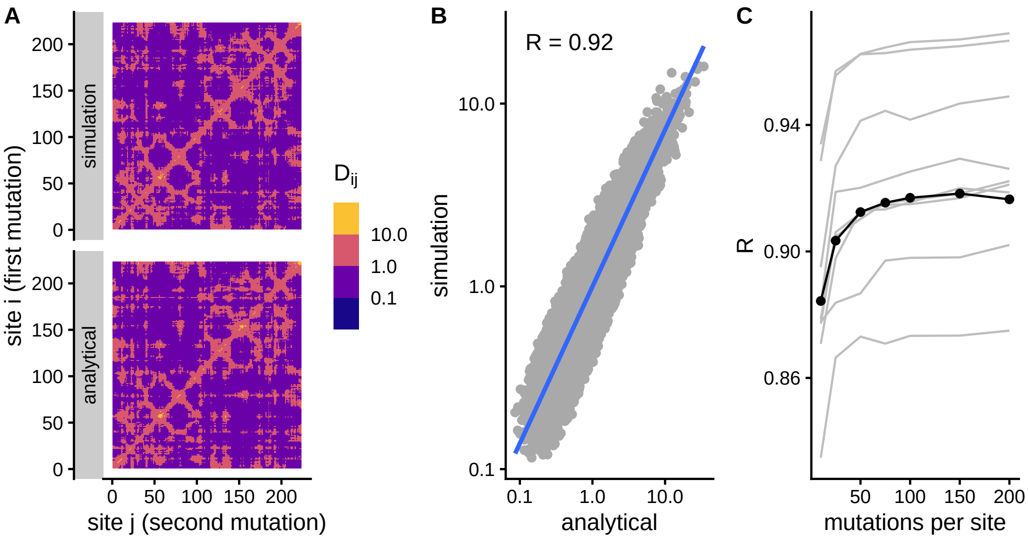

Compensation profiles, d1mcta

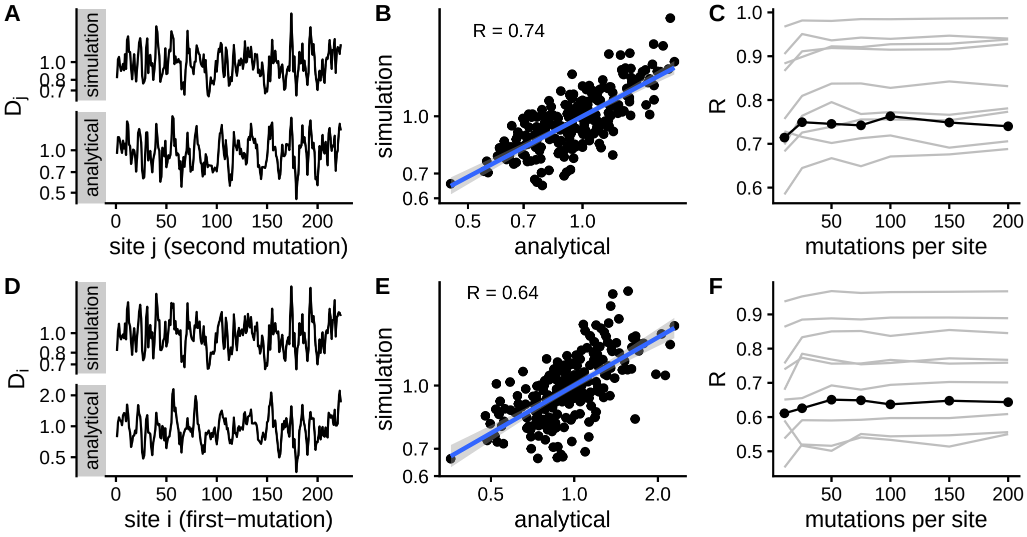

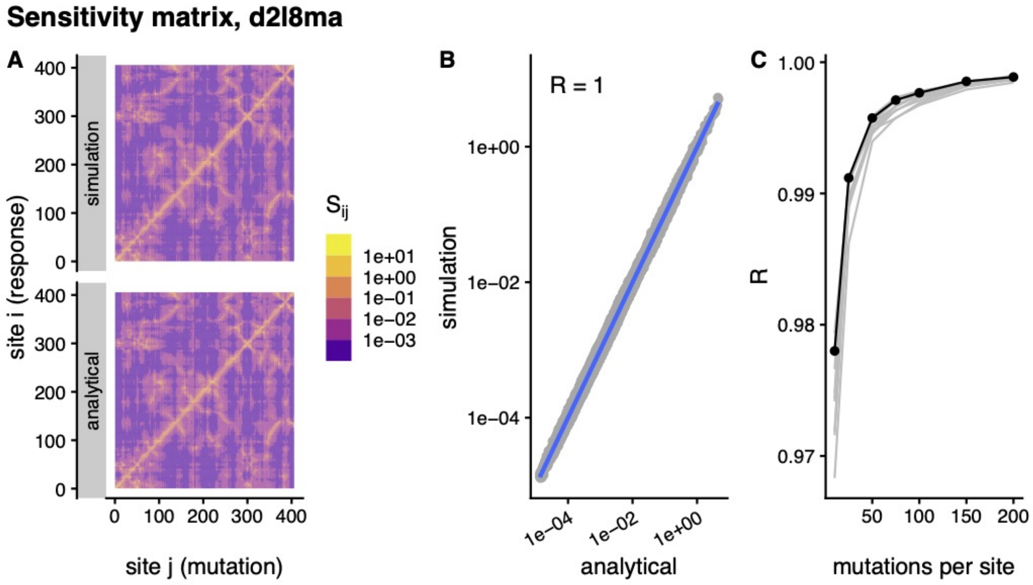

Sensitivity matrix, d2l8ma
A
B
C
site i (response)
simulation
analytical
site j (mutation)
S_{ij}
1e+01
1e+00
1e−01
1e−02
1e−03
R = 1
simulation
analytical
R
mutations per site

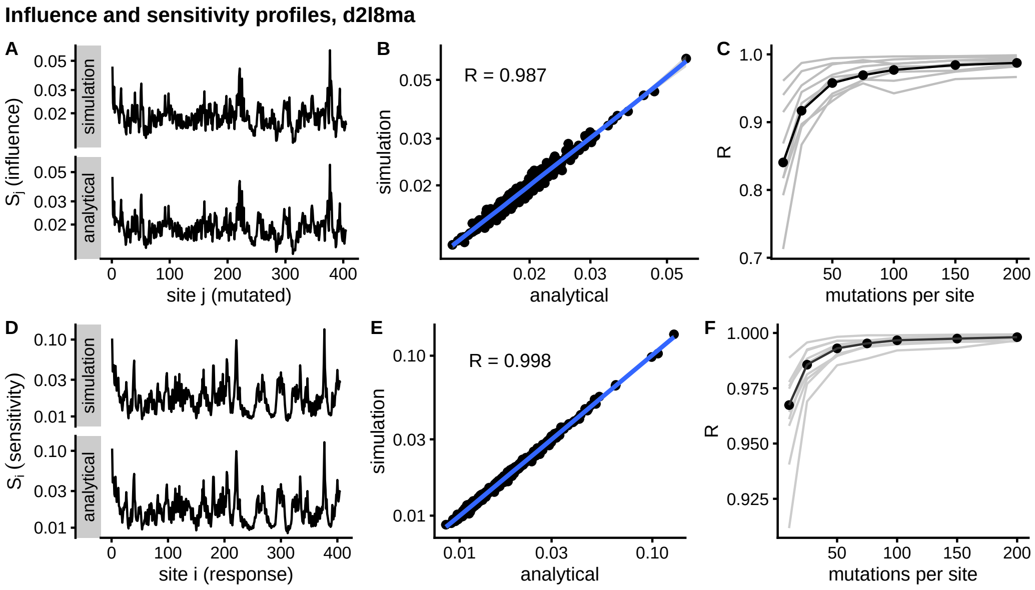

Influence and sensitivity profiles, d2l8ma
A
B
C
D
E
F
S_j (influence)
site j (mutated)
R = 0.987
R = 0.998
simulation
analytical
R
mutations per site
S_i (sensitivity)
site i (response)

Compensation matrix, d2l8ma

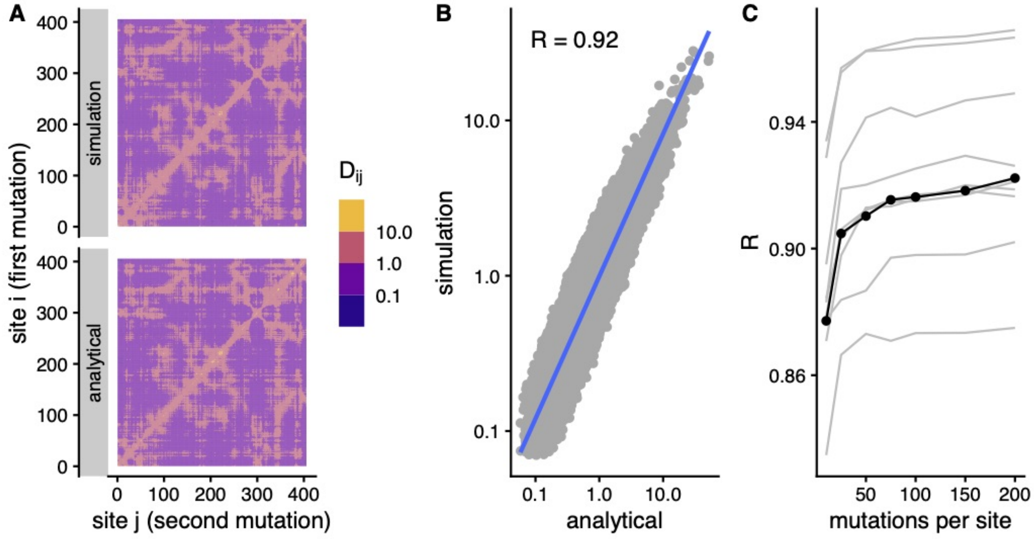

Compensation profiles, d2l8ma

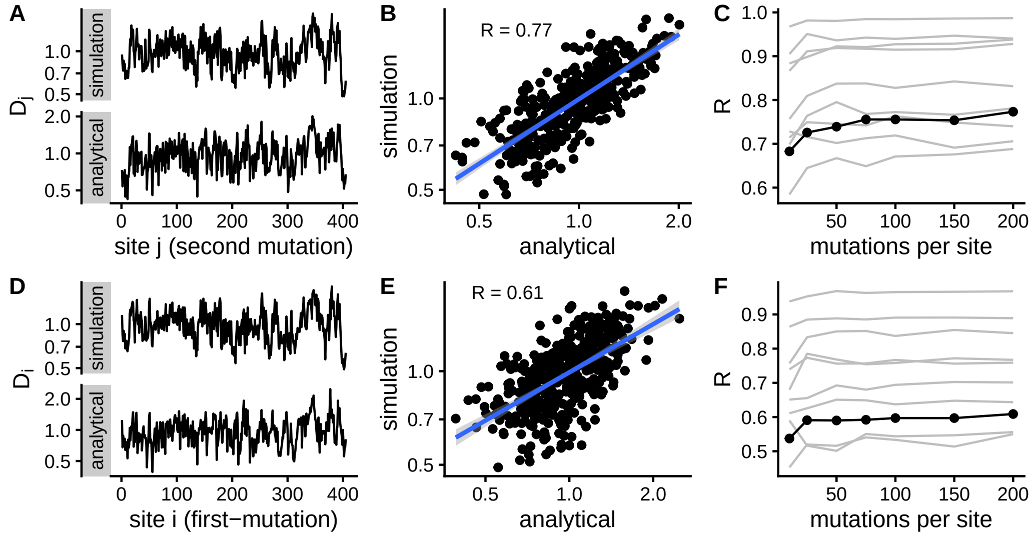

Supplement: Supplemental Information 1 [file peerj-09-11330-s001.pdf]
